# Essential Oils as Antimicrobial Agents Against WHO Priority Bacterial Pathogens: A Strategic Review of In Vitro Clinical Efficacy, Innovations and Research Gaps

**DOI:** 10.3390/antibiotics14121250

**Published:** 2025-12-10

**Authors:** Katia Iskandar, Nada Ahmed, Narayan Paudyal, Maria-Jose Ruiz Alvarez, Subramani Paranthaman Balasubramani, Danielle Saadeh, Sami Ullah Baig, Hiba Sami, Dalal Hammoudi Halat, Nebojša Pavlović, Christine Roques, Meher Rizvi, Pascale Salameh, Faten Hamed, Maarten Van Dongen

**Affiliations:** 1 Department of Biomedical Sciences, School of Pharmacy, Lebanese International University, Beirut 1105, Lebanon; faten.hamed@liu.edu.lb; 2 Faculty of Public Health-Section 2, Lebanese University, Fanar 2611, Lebanon; 3 INSPECT-LB (Institut National de Santé Publique, d’Épidémiologie Clinique et de Toxicologie-Liban), Beirut 1103, Lebanonpascalesalameh1@hotmail.com (P.S.); 4 Department of Microbiology, High Institute of Public Health, Alexandria University, Alexandria 5424041, Egypt; n.amed.aca@gmail.com; 5 National Animal Health Research Center, Nepal Agricultural Research Council (NARC), Khumaltar, Lalitpur P.O. Box 3733, Nepal; narayan.paudyal@outlook.com; 6 Research Coordination and Promotion Service, National Institute of Health (ISS), Viale Regina-Elena, 299, 00161 Rome, Italy; 7 Department of Natural Sciences, Albany State University, 2400 Gillionville Rd., Albany, GA 31707, USA; balasubramani.subramaniparanthaman@asurams.edu; 8 Department of Humanities and Social Sciences, Bahria University Islamabad Campus, Islamabad 44000, Pakistan; 9 Department of Microbiology, Jawaharlal Nehru Medical College, Aligarh Muslim University, Aligarh 202001, India; 10 QU Health Office of Assessment and Accreditation, QU Health, Qatar University, Doha P.O. Box 2713, Qatar; dhammoude@qu.edu.qa; 11 Department of Pharmacy, Faculty of Medicine, University of Novi Sad, Hajduk Veljkova 3, 21000 Novi Sad, Serbia; nebojsa.pavlovic@mf.uns.ac.rs; 12 Laboratoire de Génie Chimique, CNRS, INPT, UPS, Faculté de Pharmacie, Université de Toulouse, 31062 Toulouse, France; roques730@aol.com; 13 ACM Pharma FONDEREPHAR, 3 Rue des Satellites, Canal Biotech 2, 31400 Toulouse, France; 14 Department of Microbiology & Immunology, College of Medicine & Health Sciences, Sultan Qaboos University, Muscat 123, Oman; rizvimeher@squ.edu.om; 15 Department of Primary Care and Population Health, University of Nicosia Medical School, Nicosia 2408, Cyprus; 16 Gilbert and Rose Marie Chagoury School of Medicine, Lebanese American University, Byblos P.O. Box 36, Lebanon; 17 AMR Insights, 1017 EG Amsterdam, The Netherlands; maarten@amr-insights.eu

**Keywords:** plant essential oils, antibacterial, antimicrobial resistance, antibiotics, clinical isolates, bacteria, synergism, challenges, extraction methods

## Abstract

The rapid rise of antimicrobial resistance (AMR) has emerged as a critical global health crisis, driven by the widespread emergence of multidrug-resistant (MDR) and extensively drug-resistant (XDR) pathogens. This growing threat, coupled with the stagnation in the development of novel antibiotics, necessitates the investigation of alternative antimicrobial strategies. Plant-derived essential oils (EOs) have emerged as promising candidates due to their broad-spectrum antibacterial activity, multi-targeted mechanisms, and capacity to enhance the efficacy of existing antibiotics. Recent studies have underscored the potential of EOs in disrupting biofilms, inhibiting quorum sensing, modulating efflux pumps, and reversing resistance in a variety of bacterial pathogens, including those listed as priorities by the World Health Organization. Notably, many of these effects have been demonstrated against resistant strains isolated directly from clinical samples, thereby enhancing the translational significance of EOs. In addition to their antimicrobial properties, advances in analytical, omics-based, and microfluidic technologies have further elucidated the mechanisms of EOs and may accelerate their therapeutic development. Nevertheless, challenges such as variability in composition, lack of standardized testing protocols, and limited in vivo data continue to impede clinical application. Therefore, the aim of this scoping review is to critically examine the advances over the past decade in the antibacterial activity of plant EOs against clinical isolates, with a particular focus on their efficacy against resistant bacterial pathogens and their potential role in combating AMR.

## 1. Introduction

The global crisis of antimicrobial resistance (AMR) has reached unprecedented levels, with the World Health Organization (WHO) declaring it among the top ten public health threats facing humanity [1]. The emergence and proliferation of multidrug-resistant (MDR) and extensively drug-resistant (XDR) bacterial pathogens have severely compromised the efficacy of conventional antibiotic therapies, necessitating urgent exploration of alternative therapeutic strategies [2,3,4,5]. The declining pipeline of novel antibiotics, coupled with the accelerating pace of resistance development, has created a critical therapeutic gap that demands innovative approaches to combat resistant pathogenic bacteria [6,7,8,9,10].

To orientate research and development, the WHO has published a Bacterial Priority Pathogens List (BPPL) that identified 24 pathogens across 15 families of antibiotic-resistant bacterial pathogens as priority targets for new antibiotics development [11]. The report emphasizes Gram-negative bacteria (GNB) resistant to last-resort antibiotics, such as *Acinetobacter baumannii*, third-generation cephalosporin-resistant and carbapenem-resistant *Enterobacterales*, as well as other high-burden resistant pathogens, including *Salmonella* spp., *Pseudomonas aeruginosa*, Group A and B *Streptococci*, *Enterococcus faecium* and *Staphylococcus aureus* [11]. According to the BPPL report, the antibiotic pipeline suffers from limited innovation and global access to new and current treatments [11].

Plant essential oils (EOs) have emerged as compelling candidates in the fight against AMR, demonstrating remarkable antimicrobial properties through complex, multifaceted mechanisms of action [12,13,14,15,16,17]. Unlike conventional antibiotics that typically target single bacterial processes, EOs exhibit synergistic effects with existing antibiotics and possess the unique ability to re-sensitize multidrug-resistant bacteria to available therapies [12,18,19,20,21,22,23]. This multi-target approach, combined with their capacity to disrupt biofilm formation, inhibit efflux pumps, and modulate bacterial quorum sensing, positions EOs as promising leads for developing novel adjunctive or alternative antimicrobial strategies [10,13,14,15,16,17].

The therapeutic potential of EOs stems from their complex chemical composition, which predominantly consists of volatile secondary metabolites including terpenes, terpenoids, and aromatic compounds [24,25,26]. These bioactive components, particularly phenolic compounds such as thymol, carvacrol, and eugenol, demonstrate potent antimicrobial effects against a broad spectrum of bacterial pathogens, including WHO-priority pathogens [13,14,27,28,29]. The natural origin and multi-component nature of EOs contribute to their reduced likelihood of inducing rapid resistance development compared to single-molecule antibiotics [30,31].

Despite extensive in vitro research demonstrating the promising antimicrobial properties of EOs, significant knowledge gaps remain regarding their clinical translation. Multiple systematic reviews have identified critical limitations, including insufficient data on in vivo bioavailability, tissue distribution, selectivity, stability, and comprehensive safety profiles required for clinical practice [10,13,14,15,16,17,32]. These limitations, coupled with concerns about complex regulatory processes, potential resistance development, and possible antagonistic interactions with conventional antibiotics, have hindered the progression of EOs from laboratory bench to clinical bedside [8,9,33,34].

The past decade has witnessed substantial advances in EO research, with increased focus on their activity against clinical isolates and exploration of their potential as lead compounds for antimicrobial drug development [15,35,36,37,38,39,40,41,42,43,44]. However, a comprehensive analysis of recent progress specifically examining EOs’ antibacterial efficacy against resistant clinical pathogens, while addressing the challenges impeding their clinical implementation, remains lacking in the current literature.

Therefore, this review aims to critically examine advances during the past decade (2014–2024) in the antibacterial activity of plant EOs against clinical isolates, with particular emphasis on their efficacy against resistant bacterial pathogens. Furthermore, we explore the current challenges and recent technological advances that may facilitate the translation of EOs into viable therapeutic options resistant bacteria. By integrating current evidence and highlighting key research gaps, this review seeks to provide a comprehensive framework for future research directions and clinical applications of EOs in the global fight against AMR.

## 2. Search Strategy

The scoping review is based on 48 studies included according to their thematic consistency and relevance to the review objective. Searched databases included Web of Science, PubMed, Google Scholar, and grey literature, from 2014 to 2024.

The search strategy consisted of four main concepts: “essential oils”, “antibacterial use” and “antibiotic resistance” and “clinical isolates”. Relevant keywords and Medical Subject Headings (MeSH) terms were combined using Boolean operators to capture the pertinent literature. The initial search results were screened based on titles and abstracts, and only relevant studies tackling the EOs antibacterial activity against clinical isolates were included in the review.

## 3. The Antimicrobial Effect of Essential Oils

### 3.1. Mechanisms of Antimicrobial Activity of Essential Oils

Plant EOs exert antimicrobial activity through multiple concurrent mechanisms, including membrane disruption, biofilm inhibition, quorum sensing (QS) interference, and suppression of antimicrobial resistance determinants such as efflux pumps and β-lactamases [45]. This multi-target activity distinguishes EOs from conventional single-target antibiotics and may explain the limited development of bacterial resistance observed in vitro.

#### 3.1.1. Disruption of Biofilm Formation

Biofilms, structured bacterial communities encased in a self-produced matrix, significantly contribute to pathogenesis and antibiotic resistance [46,47].

Some EOs act as antibiofilm agents by disrupting formation processes and dismantling mature biofilms across a broad spectrum of GNB and Gram-positive bacteria (GPB) [37,48]. During biofilm formation, EOs disrupt planktonic cells before attachment through cell wall [48,49] or membrane disruption [50], reactive oxygen species (ROS) generation [51] and interference with energy production [52]. They also alter surface properties such as hydrophobicity and charge [53], preventing bacterial cells attachment [38] or microcolony formation [54]. Additionally, EOs affect virulence factors directly by regulating gene expression [55] or by interacting with proteins involved in virulence processes [48,56]. The latter effects occur via intracellular signaling pathways such as C-AMP [54] or c-di-GMP [57,58,59,60,61,62].

In vitro studies have demonstrated that cassia oil, cinnamon oil, tea tree oil, and palmarosa oil effectively reduce biomass and bacterial cells within established *P. aeruginosa* biofilms, in some cases outperforming antibiotics such as ciprofloxacin [48]. Oregano oil containing 72.3% carvacrol completely eradicated *S. aureus* and *P. aeruginosa* within biofilms, with no bacterial resistance development observed after 20 passages [35].

In clinical settings, EOs and their compounds could be valuable at biocidal concentrations by reducing bacterial colonies and destroying biofilm matrix [63]. This finding is relevant in preventing biofilm formation on clinical surfaces and devices, particularly catheters. Jafri and Colleagues (2014) evaluated the anti-biofilm activities of various EOs and active compounds against antibiotic-resistant *S. aureus* strains, reporting that eugenol and thyme oil exhibited concentration-dependent biofilm inhibition [63]. At biocidal concentrations of 4–12.8%, eugenol inhibited biofilm formation of antibiotic-resistant *S. aureus* strains by nearly 90%, while thyme oil demonstrated a maximum reduction of 88.7% in biofilm formation [63].

#### 3.1.2. Modulation of Quorum Sensing Activity

QS is a bacterial communication system responding to population density changes, regulating virulence factors and biofilms protection against innate immune system [64,65,66,67,68,69]. The process involves autoinducers (AIs): GPB predominantly use autoinducing peptides (AIPs), while GNB employ acyl-homoserine lactones (AHLs or AI-1) [48,70], with both types producing AI-2 (furanosyl borate diester) for intra- and interspecies communication [46,56].

Recent in vitro studies have shown that certain EOs and their components modulate QS-related genes expression, inhibiting biofilm formation and virulence factor production [48,68,70,71,72,73,74,75,76]. *Melaleuca bracteate* and *Artemisia princeps* EOs regulated QS genes (*lasI*, *lasR*, *rhlI*, and *rhlR)* in *P. aeruginosa* [71] and virulence genes (*mecA*, *sea*, *agrA*, and *sarA*)in MRSA, respectively [72]. Clove oil, peppermint oil, and menthol exhibit anti-QS activity by interfering with the AHL-QS signaling cycle, inhibiting the LasR/RhlR regulatory system in *P. aeruginosa* [74,75], and attenuating virulence factors production, including pyocyanin, elastase, chitinase, and proteases. Notably, monoterpenes and phenylpropanes (thymol, carvacrol, and linalool) demonstrated anti-QS effects at sub-MICs, suggesting a specific interference with QS systems rather than general antimicrobial activity [76].

#### 3.1.3. Cellular Targets: Membrane Activity and Genetic Material

Primary antibacterial mechanism of plant EOs involves the disrupting bacterial cytoplasmic membrane integrity and function [48]. EOs alter membrane fluidity and permeability, interfere with transport proteins, and influence fatty acid composition [48,70]. They also impact cell division, cell wall structure, morphology, respiration, ion transport, and energy balance [48].

Specific mechanisms include forming multilamellar structures within membranes, disintegrating outer membranes and lipopolysaccharides (LPS) in GNB, and inducing cell lysis [48]. Some EOs eliminate R-plasmids contributing to AMR spread [77]. For example, cinnamic acid interacts with the FtsZ protein to inhibit cell division and elongation [78], while thyme EOs alter cellular protein composition similarly to streptomycin and gentamicin [79]. Cinnamaldehyde, the predominant compound in cinnamon EOs, interacts with β-galactosidase, an essential bacterial enzyme, leading to conformational changes and significant activity decline [80].

EOs generally show greater efficacy against GPB due to their hydrophobic nature allowing easier penetration of the Gram-positive peptidoglycan layer [81,82], though diverse mechanisms of EOs can potentially overcome the protective lipopolysaccharide barrier in GNB by increasing permeability and enhancing antibiotic uptake [52,82].

EOs have also demonstrated the ability to indirectly affect microbial genetic material, primarily through oxidative stress and the induction of epigenetic changes [83]. EOs derived from *Clinopodium nepeta*, *Origanum vulgare*, and *Foeniculum vulgare* showed the most pronounced inhibitory effects on bacterial growth across various cell lines by inducing epigenetic modifications, such as methylation at adenine and cytosine residues [83]. ROS generated by EOs negatively regulate gene expression involved in motility, adherence, cellular aggregation, and exopolysaccharide production in *E. coli* and *S. aureus* [84]. However, genomics and proteomics studies suggest EOs do not directly damage microbial DNA but exert their antimicrobial effects through complex genomic interactions [16,85,86,87].

#### 3.1.4. Dual Pro-Oxidant and Antioxidant Activities

The dual pro-oxidant and antioxidant activities of EOs contribute to their complex antibacterial effects and potential to combat AMR [88]. EOs generate ROS or inhibit bacterial antioxidant mechanisms [88], inducing oxidative stress that damages cells, inhibits biofilm formation, and disrupts essential cellular functions [52,73,88,89]. *Cinnamomum verum* EOs elevate ROS levels in *K. pneumoniae*, causing leakage of intracellular contents and interfering with energy production and cell wall synthesis [89]. *Lavandula angustifolia* EOs combined with meropenem increase ROS levels in *K. pneumonia cells*, leading to oxidative damage and membrane disruption [52]. *Rosmarinus officinalis* and *Myrtus communis* EOs reduce catalase production jeopardizing bacterial adaptation to oxidative stress [90]. Bowbe and Colleagues (2023) showed that the anti-staphylococcal activity of *Rosmarinus officinalis* and *Myrtus communis* EOs involved modulating the bacterium antioxidant responses by reducing catalase production and jeopardizing the bacterial adaptation to oxidative stress induced by the EOs [90].

In hosts, EOs exhibit antioxidant properties protecting host cells from oxidative damage and modulating immune responses, supporting the ability to fight infections and reduce inflammation [64,65,66,67,68,88].

#### 3.1.5. Inhibition of Resistance Mechanisms

##### Efflux Pump Effect

Efflux pumps are membrane proteins that expel antibiotics, maintaining subtherapeutic concentrations [91]. *Cinnamomum zeylanicum* EO demonstrated potent antibacterial activity against MDR *P. aeruginosa* by significantly downregulating the expression of *mexA* and *mexB* efflux pump genes, enhancing existing antibiotic efficacy [92]. Mentha EOs constituents effectively inhibit DNA gyrase and the MDR *E. coli* AcrB-TolC efflux pump [93], while thyme extract inhibits *norA* gene expression encoding a multidrug efflux pump protein in *S. aureus* clinical isolates [94,95] Oregano and thyme EOs inhibited the *pmrA* gene expression in fluoroquinolone-resistant *S. pneumoniae* [96].

##### β-Lactamase Inhibition

Various EOs have demonstrated promising β-lactamase inhibition abilities. *Piper tuberculatum* EOs exhibited activity comparable to sulbactam in *S. aureus* [37], while clove EOs inhibited β-lactamases across *S. aureus*, *E. coli*, *K. pneumoniae*, and *P. aeruginosa* [97]. Terpinolene in various EOs showed effective β-lactamase inhibition [98], and ginger EOs demonstrated a synergistic effect with cefepime against β-lactamases of *E. coli* causing urinary tract infection (UTI) [99].

#### 3.1.6. Additional Anti-Virulence Mechanisms

EOs target and inhibit bacterial toxins from species such as *Klebsiella pneumoniae* [100], *E. coli* [101], and *P. aeruginosa* [102]. Clove, cinnamon, and oregano oils reduced *Campylobacter jejuni *(*C. jejuni*) attachment and invasion of intestinal epithelial cells [103]. Regarding antimotility effects, carvacrol, found in oregano EOs, inhibited flagellin production in *E. coli* E157:H7, rendering it non-motile [104], while trans-cinnamaldehyde, carvacrol, and eugenol inhibited *C. jejuni* motility, and thymol inhibited *P. aeruginosa* motility [102,103].

### 3.2. Antibacterial Efficacy of Selected Plant Essential Oils Against Resistant Clinical Pathogens

EOs are complex volatile compounds derived from various plant parts, including leaves, flowers, fruits, seeds, bark, and root [10]. Their chemical composition primarily consists of terpenes (monoterpenes and sesquiterpenes), terpenoids (oxygenated derivatives such as phenols, alcohols, aldehydes, ketones, and ethers), and aromatic compounds [25,105]. Typically colorless to pale yellow, EOs possess strong aromatic properties and are poorly water-soluble but highly soluble in organic solvents [106,107].

The major bioactive components (20–70%) of EOs exhibit potent antimicrobial effects, with phenolic compounds being the most effective, followed by terpenes and non-aromatic alcohols [13,105,108]. Oxygenated terpenes demonstrate more potent activity than their hydrocarbon counterparts [108]. The synthesis of these secondary metabolites occurs via pathways such as the mevalonic acid and methylerythritol phosphate pathways [107]. Their antimicrobial efficacy stems from their complex composition, with notable potency observed in the Lamiaceae family due to high thymol and carvacrol content [109]. Beyond direct antibacterial effects, some EOs can sensitize resistant bacteria to conventional antibiotics [110], making them promising candidates against AMR. This section reviews recent studies on the antibacterial effects of different EOs and is summarized in Table 1.

#### 3.2.1. Cinnamon Essential Oils

Several types of cinnamon essential oils are known, which vary based on the plant species and the part of the plant used for extraction. The two most common species used for essential oil production are *Cinnamomum verum* (true cinnamon) and *Cinnamomum cassia* (Chinese cinnamon) [89,111,112,113,114,115,116,147,148,149,150,151,152].

Cinnamon EOs and their main component, cinnamaldehyde, have shown notable antibacterial, anti-QS, and antibiofilm activities against numerous bacterial pathogens. This includes antibacterial and anti-biofilm activity against *Escherichia coli* (*E.coli*) carrying *pks* gene isolated from patients with colon cancer and inflammatory bowel disease, as well as healthy individuals, with MICs of 32 and 0.03 μg/mL for the EO and cinnamaldehyde respectively [111]. Cinnamon EOs also exhibited antibacterial and anti-efflux activity against pan- and extensively drug-resistant *Pseudomonas aeruginosa* (*P. aeruinosa*) isolated from human burns and urine samples with MICs ranging from 0.0562–0.225 μg/mL, while the minimum bactericidal concentrations (MBC) were 0.1125–0.225 μg/mL [112]. In another study, cinnamon, thyme, and eucalyptus EOs were tested against multi-drug-resistant (MDR) colistin-resistant Gram-negative bacteria (GNB) isolated from cancer patients, and exhibited MICs of 4.88–312.5 μg/mL [113]. Cinnamon oil demonstrated the highest efficacy against colistin-resistant *Proteus mirabilis* and *E. coli* compared with thyme and eucalyptus EOs [113]. The treatment with cinnamon oil led to a significant downregulation of the *mcr-1* gene, associated with colistin resistance, by 20 to 35 fold [113].

In one study, eugenol, the most abundant EOS component, was shown penetrate and disrupt the phospholipid bilayer of the cell membrane, altering its fluidity and permeability and ultimately leading to cell death in some pathogens including *P. aeruginosa*, *Staphylococcus aureus*, and *K. pneumoniae* [150].

*Cinnamomum cassia* (*C. cassia*) EOs showed synergistic potential with various antibiotics against various GNB and GPB [151]. Similarly, the EOS exhibited antibacterial activity against carbapenemase-producing *K. pneumoniae* and *Serratia marcescens* (*S. marcescens)* strains isolated from human specimens [115]. T The major components of these EOs were cinnamaldehyde (87.6%), α-humulene (3.1%), γ-element (2.5%), borneol (1.5%), cinnamic acid (0.7%), benzaldehyde (0.5%), and eugenol (0.4%) [115]. The EOs showed a MIC of 281.25 μg/mL and a FICI of 0.006 when combined with polymyxin B, indicating a strong synergistic effect [115]. In an extremely drug-resistant *K. aerogenes*, with 17 resistance genes identified by whole genome sequencing, and conferring resistance to carbapenems (*bla*_KPC-2_), cephalosporins (*bla*_CTX-M-15_), aminoglycosides, chloramphenicol, trimethoprim, fluoroquinolones, sulfamethoxazole, and tetracycline, *C. cassia* EOs exhibited antibacterial activity with a MIC of 17.57 μg/mL, highlighting the potential of this EO against MDR *Klebsiella* [115].

#### 3.2.2. Clove Essential Oils

Eugenol EOs, derived from *Syzygium aromaticum* buds, demonstrated significant antibacterial and antibiofilm properties against MDR *Helicobacter pylori* clinical isolates, with MIC values ranging from 23.0 to 51 μg/mL [117]. In another study, clove EOS showed antimicrobial and virulence-modulating effects against *Campylobacter jejuni*, a prevalent foodborne pathogen [118]. Proteomic and transcriptomic analyses further revealed that clove EOs disrupted the expression of multiple virulence-related genes, including those involved in flagellar synthesis, protein energy binding 1 and 4 (PEB1 and PEB4), lipopolysaccharide biosynthesis, and serine protease production. The induced stress responses significantly impacted motility, highlighting the dual role of clove EOs as an antimicrobial agent and a modulator of virulence factors in *C. jejuni* [118].

Additional studies examined the potential of clove and thyme EOs to be incorporated into chitosan-coated nanoemulsions (NEs) for intranasal drug delivery to treat brain infections [119]. The intranasal route presents an alternative to traditional delivery methods by bypassing the blood-brain barrier (BBB) through the olfactory region [119]. The chitosan-coated NEs exhibited significant efficacy against methicillin-susceptible *S. aureus* and MDR GNB, including carbapenem-resistant strains of *A. baumannii* and *K. pneumoniae* [119].

#### 3.2.3. Eucalyptus Essential Oils

*Eucalyptus camaldulensis* EOs demonstrated significant antimicrobial efficacy against MDR *A. baumannii* isolates, with MIC values ranging from 0.5 to 2 μL/mL [20]. The oils, rich in spatulenol, cryptone, and p-cimene, exhibited remarkable synergistic effects when combined with conventional antibiotics, particularly polymyxin B [20]. Similarly, the antibacterial activity of eucalyptus (*Eucalyptus globulus*), in addition to the tea tree (*Melaleuca alternifolia*), clove (*Syzygium aromaticum*), and cinnamomum (*Cinnamomum zeylanicum*) EOS, were evaluated against GPB and GNB [59]. Results showed that 64.43% of *P. aeruginosa*-producing biofilm and 80.32% of non-biofilm-producing strains showed sensitivity to eucalyptus EOs [59]. In addition, 54.16% of S. aureus biofilm producers and 68.75% of non-biofilm producers were sensitive to Eucalyptus EOs [59].

#### 3.2.4. Geranium Essential Oils

The EOs extracted from *Pelargonium graveolens*(geranium) demonstrated antibacterial efficacy against drug-resistant strains of *E. coli*, *Citrobacter freundii*, *Enterobacter sakazakii*, *E. cloacae*, *P. mirabilis*, and *P. aeruginosa* isolated from clinical wounds [124]. The findings suggest that geranium EOs can be a topical treatment for recurrent drug-resistant wound infections, predominantly those due to *E. coli* [124]. Kafa (2022) studied in vitro the antibacterial and antibiofilm activity of *Pelargonium graveolens* L., *Rosemary officinalis* L., and *Mentha piperita* L. against extensive drug-resistant (XDR) colistin-resistant and colistin-susceptible *A. baumannii* clinical strains [40]. The EOs sub-MICs exerted a biofilm inhibitory effect ranging from 48% to 90% of the tested clinical isolates [40]. Pelargonium EO and rosemary EO antibacterial activity ranged from 5 to 20 μL/mL [40], and a potent synergistic effect with colistin allowing for a 2- to 32-fold reduction in colistin MIC when used with these EOs [40].

#### 3.2.5. Lemongrass Essential Oils

Prophylactic application of lemongrass EOs to *K. pneumoniae*, *P. aeruginosa*, and *S. epidermidis* isolates from chronic rhinosinusitis patients effectively led to the disruption of biofilm formation across all bacterial isolates and enhanced *P. aeruginosa* swarming motility while maintaining cellular compatibility with human tissue [126].

In a comparative analysis of twelve EOs, *Cymbopogon citratus* EOs demonstrated superior antibacterial efficacy against bacterial species implicated in pitted keratolysis, a cutaneous infection predominantly affecting plantar pressure points [127]. The investigation revealed remarkably low MIC and MBC values (0.1 mg/mL) against *Kytococcus sedentarius*, *Dermatophilus congolensis*, and *Bacillus thuringiensis* [127]. In a comprehensive analysis of 51 clinical isolates comprising 18 sequence types, six EOs, including lemongrass, lavender, marjoram, peppermint, tea tree, and rosewood, exhibited potent antimicrobial properties against *Burkholderia cepacia* complex [41]. Among these, lemongrass and rosewood oils demonstrated superior efficacy, with remarkably low MIC50 and MIC90 values of 0.5% and 1%, respectively [41].

#### 3.2.6. Mentha Essential Oils

The bactericidal activity of peppermint EOs against MDR and XDR strains of *S. aureus*, *E. coli*, *K. pneumoniae*, *P. mirabilis*, *P. aeruginosa*, and *A. baumannii* [128]. Results demonstrated a bactericidal effect, with a MIC ranging from 20 mg/mL for *S. aureus*, *E.coli*, and *P. mirabilis* to 40 mg/mL for *K. pneumoniae*, *P. aeruginosa*, and *A. baumannii* [128]. The MBC was equal to the MIC for most strains, except for *P. aeruginosa*, where the MBC was twice the MIC [128].

*Mentha longifolia* demonstrated significant potential in combating AMR in *A. baumannii* with overexpression of efflux pump genes [42]. In this study, *M. longifolia* EOs reduced the MICs of ciprofloxacin and imipenem by 4- and 8-fold, respectively, while menthol, its active ingredient, decreased imipenem resistance by up to 16-fold [42].

#### 3.2.7. Oregano Essential Oils

The EOs derived from various *Origanum* species, particularly *Origanum vulgare* (oregano) and *Origanum compactum*, have demonstrated potent antibacterial and anti-biofilm activities against various bacterial pathogens. For instance, *Origanum vulgare* EOs showed significant inhibitory effects against carbapenem-resistant *K. pneumoniae*, *S. marcescens*, and *A. baumannii* isolated from human rectal swab, urine, and nasal swab, respectively [129]. The MICs were as low as 0.059% *v*/*v* for *K. pneumoniae* and *S. marcescens* and 0.015% *v*/*v* for *A. baumannii* [129]. Similarly, *Origanum vulgare* EOs alone or in combination with polymyxin B inhibited MDR *A. baumannii* clinical isolates [130]. Additionally, oregano and thyme EOs effectively inhibited biofilm formation in uropathogenic *E. coli* O6:H1 strain CFT073 (UPEC) [131]. Similarly, *Origanum onites* EOs showed antibacterial effect against ESBL *E. coli* clinical isolates [132].

The potential antibacterial activity of oregano EOs against MDR bacteria was also demonstrated, particularly in trauma-associated wound infections [35]. Oregano EOs exhibited significant antibacterial activity against 11 MDR clinical isolates, including *A. baumannii*, *P. aeruginosa*, and MRSA, with MICs ranging from 0.08 mg/mL to 0.64 mg/mL. Resistance to oregano oil was not detected even after 20 passages in the presence of sublethal doses [35]. In a study where multiple EOs were tested, oregano oil also showed a MIC/MBC value up to 64 times lower than ethylic alcohol, particularly against MRSA [153].

Other investigations included the evaluation of the antibacterial and anti-biofilm activities of wild oregano, garlic, and black pepper EOs against *C. difficile* clinical isolates, which frequently exhibit multidrug resistance [133]. Wild oregano EOs exerted potent inhibitory activity with concentrations ranging from 0.02–1.25 mg/mL and potent bactericidal activity with concentrations varying from 0.08 to 10 mg/mL [133]. In another study, various EOs including oregano were tested against 32 strains of erythromycin resistant *Streptococcus pyogenes* isolated from children with pharyngotonsillitis [43]. While oregano and thyme EOs showed moderate antibacterial activity, their major component carvacrol demonstrated the strongest effects, killing bacteria within an hour and showing no development of resistance [43]. When carvacrol was combined with erythromycin, it significantly reduced the required amount of erythromycin by 2 to 2,048 times using checkerboard assays in many strains, with the synergistic effect particularly strong in bacteria that only expressed resistance when exposed to erythromycin [43].

#### 3.2.8. Rosemary Essential Oils

*Rosmarinus officinalis* L. EOs exhibited potent antibacterial activity against *S. aureus *(ATCC 29737), *K. pneumoniae* (ATCC 10031), and *Proteus vulgaris *(PTCC 1182) isolated from urine specimens of patients suspected of UTI [134]. The predominant EO components were 1,8-cineole (17.16%) and α-pinene (16.95%) [134]. Similarly, the antibacterial and antibiofilm activities of *Rosmarinus officinalis*, *Origanum majorana*, and *Thymus zygis* EOs were evaluated against *E. coli* isolated from patients with UTI [135]. *Rosmarinus officinalis* EOs were most effective against biofilm formation, inhibiting 77.27% of the isolates [135].

#### 3.2.9. Tea Tree Essential Oils

Numerous studies showed that *Melaleuca alternifolia* (tea tree) EOs exhibits promising antibacterial and antibiofilm activities against MDR clinical bacterial isolates. Terpinen-4-ol, the primary EOs constituent, significantly inhibited MRSA biofilm formation on glass by 73.70%, reduced violacein production in *C. violaceum* by 69.3% at a MIC value of 0.048 mg/mL, and decreased swarming motility in *P. aeruginosa* PAO1 by 33.33% [60]. These findings suggest that tea tree EOs, at very low doses, exhibit promising anti-QS and antibiofilm activities, warranting further investigation as a potential alternative treatment for MRSA infections [60]. In another study, inhaled tea tree EOs displayed synergistic effects with tobramycin against clinical cystic fibrosis-associated MDR *P. aeruginosa* isolates [136], with a fractional biofilm eradication concentration index (FBECI) of 0.42 [136]. Tea tree EOs nanoemulsion showed comprehensive antimicrobial activity, eliminating planktonic and biofilm-associated bacterial forms, alongside a capacity to reduce bacterial virulence factors and induce the production of ROS within bacterial cells [137].

#### 3.2.10. Thyme Essential Oils

Thymus EOs extracted from various thymus plant species, including *Thymus vulgaris* (thyme), *Thymus zygis*, *Thymus defenses*, and *Thymus pallescens*, have demonstrated remarkable antibacterial, anti-biofilm, and anti-QS properties against a diverse array of bacterial pathogens. Notably, *Thymus pallescens* EOs emerged as the most potent antimicrobial agent against clinical strains of *K. pneumoniae*, *E. coli*, and *S. aureus*., exhibiting large inhibition zones (up to 50 mm against *S. aureus*), low MICs (0.16–0.63 mg/mL), and potent biofilm eradication at 24- and 60-min post-exposure [138].

*Thymus daenensis* and *Origanum vulgare* EOs demonstrated the ability to modulate efflux pumps in fluoroquinolone-resistant *S. pneumoniae* clinical isolates [96]. At sub-inhibitory concentrations (MIC/2), both EOs caused significant downregulation of the *pmrA* gene, involved in efflux pump activity and antibiotic resistance [96]. The major EOs in *Thymus daenensis* were carvacrol, γ-terpinene, and α-terpinene, and pulegone, 1,8-cineole, and borneol in *Origanum vulgare* EOs. Similarly, a study evaluated *Thymus daenensis* EOs, *Satureja hortensis* EOs, and *Origanum vulgare* EOs’ antimicrobial activities on planktonic growth, biofilm formation, QS, and the competence system of *S. pneumoniae* [139]. The MICs against planktonic *S. pneumoniae* ranged from 0.625–1.25 μL/mL for *Thymus daenensis*, 2.5 μL/mL for *Satureja hortensis*, and 2.5–10 μL/mL for *Origanum vulgare* EOs, with *Thymus daenensis* exhibiting the most potent anti-biofilm activity [139]. The *luxS* and *pfs* genes involved in QS were downregulated following treatment with MIC/2 concentrations of Thymus and Satureja EOs [139]. Further research studied *Cinnamomum verum*, *Origanum majorana*, *Thymus vulgaris*, and *Eugenia caryophyllata* EOs against MDR bacterial clinical isolates, including *E. coli*, *K. pneumoniae*, *A. baumanii*, *P. aeruginosa*, *Citrobacter freundii*, *Klebsiella oxytoca*, *Salmonella enteritidis*, *S. Typhimurium*, *Salmonella zinzibar*, *Salmonella livingstone*, *Salmonella derby*, and *Salmonella heidelberg*, *Corynebacterium striatum* and *S. aureus* [44]. Clove EOs, thyme, and cinnamon EOs exhibited significant reductions in violacein production (84.13%, 99.41%, and 91.68%, respectively), while marjoram EOs had the lowest effect (9.09%). *Cinnamon verum* EOs and *Thymus vulgaris* EOs demonstrated remarkable antibacterial activity, with inhibition zone diameters above 20 mm [44]. With marjoram EOs showing limited antibacterial efficacy, these findings suggest that cinnamon, clove, and thyme are promising antimicrobial, anti-biofilm, and anti-QS agents against MDR bacterial pathogens [44]. In an additional study, the potent antimicrobial activity of six essential oils, including that of *Thymus vulgaris* (thyme), was demonstrated against both environmental and the EOs of thyme, clove and oregano showed efficacy against ciprofloxacin-resistant strains, suggesting potential therapeutic applications for treating MDR Bcc infections, especially in immunocompromised and cystic fibrosis patients where such infections pose a significant clinical challenge [140].

#### 3.2.11. Other Essential Oils

In addition to the plant EOs described above, research has demonstrated diverse antimicrobial properties of other EOs against clinically significant bacteria.

The chemical composition and antimicrobial properties of essential oils from four *Piper* species were studied [154]. The results demonstrated that *Piper nigrum* (*P. nigrum)* and white pepper were rich in monoterpenes (87.6% and 80% respectively), while *Piper cubeba* was distinctive for its high aromatic content (59%). Notably, all oils exhibited inhibitory activity against *H. pylori*, with *Piper longum* showing the most potent effect (MIC 1.95 μg/mL, equivalent to clarithromycin) [154]. *Nectandra megapotamica* EOs demonstrated potent synergistic antibacterial effects with imipenem against carbapenem-resistant *A. baumannii* [141]. The FICI of 0.156 indicated synergism, with the oil reducing imipenem’s MIC 8-fold while its own MIC decreased 32-fold in combination [141]. The proposed mechanisms include bacterial outer membrane destabilization, facilitating antibiotic entry, and membrane permeabilization [141]. *Ocimum basilicum* (basil) and *Salvia officinalis* (sage) EOs inhibited *P. aeruginosa* clinical isolates biofilm formation by up to 99.9% compared with controls and significantly reduced biofilm production, motility patterns and pyocyanin production [143]. Other studies investigated the potential of *Salvia* EOs, particularly *S. fruticosa*, as efflux pump inhibitors in tetracycline-resistant *S. epidermidis*. This EO effectively reduced tetracycline MIC, decreased antibiotic efflux, and downregulated *tet(K)* gene expression [142]. These findings suggest potential therapeutic applications in combination therapy, where EOs could help restore bacterial susceptibility to antibiotics through efflux pumps inhibition [142]. The EOs from *Pituranthos chloranthus*, *Teucrium ramosissimum*, and *Pistacia lentiscus* were tested against MDR strains, including ESBL-producing *E. coli*, ceftazidime-resistant *A. baumannii*, and MRSA [12]. Results indicated synergism with ofloxacin and novobiocin against ESBL-producing *E. coli* and showed potent antibacterial effectiveness against MRSA compared with Gram-negative tested strains [12].

Investigation of *the Syzygium cumini* EOs effect showed moderate activity against *E. coli* ATCC 25922 and potentiated effect of antibiotics, including gentamicin, erythromycin, and norfloxacin, suggesting possible synergism [144].

Studies also highlighted the synergistic potential of *Mentha pulegium* and *Artemisia herba alba* EOs in combination with antibiotics against MDR GPB and GNB bacteria [145]. MIC values ranged from 1.2 to 9.4 µL/mL for *M. pulegium* EOs and 1.2 to 4.7 µL/mL for *A. herba alba* EOs [145]. Both EOs were effective against *Listeria innocua*, *S. aureus* (including MRSA) from pus, and *E. coli*, *P. aeruginosa*, and imipenem-resistant *A. baumannii* from catheters [1]. The strongest synergistic effects were observed for *M. pulegium* EOs with amikacin against imipenem-resistant *A. baumannii* and for *A. herba alba* EOs with cephalexin against MRSA [145].

*Nigella sativa* EO demonstrated dose-dependent antibacterial activity against multidrug-resistant *Staphylococcus aureus* isolates from diabetic wounds, with 8 of 19 isolates (42%) showing susceptibility at concentrations ranging from 200 mg/ml to undiluted oil. The remaining 11 isolates (58%) exhibited complete resistance to all tested oil concentrations [120]. In another study, *N. sativa* EO displayed potent antimicrobial effects against clinical strains of methicillin-resistant S. aureus and methicillin-resistant coagulase-negative Staphylococci, achieving inhibition at remarkably low minimum inhibitory concentrations below 0.25–1.0 µg/mL [121].

Coriander EOs along with Cinnamomum (*Cinnamomum cassia*), and *Ziziphora hispanica* EOs were tested against different susceptible and resistant bacterial phenotypes isolated from patients with UTI [123]. In this study, only cassia EOs showed a potent antibacterial activity with a MIC< 5mg/ml [123]. Coriander EOs demonstrated activity against MDR uropathogenic E. coli (UPEC) strains by inducing structural modifications in bacterial cells and reducing the gentamicin MIC, indicative of a synergistic effect [122].

Ginger EOs were also effective against *P. aeruginosa* producing extended-spectrum β-lactamase (ESBL) enzymes isolated from burn wounds with MIC and MBC values of 1.5 mg/mL and 2.0 mg/mL, respectively [125]. The antibacterial mechanism of ginger EOs in combination with cefepime demonstrated considerable efficacy against beta-lactamase-producing UTI E. coli isolates [99]. The synergistic action operates through ginger EOs’ ability to inhibit beta-lactamase enzymes, particularly those encoded by blaTEM genes, thereby enhancing cefepime’s antibacterial activity [99]. Finally, the antibacterial and anti-biofilm activity of four EOs, including *Melaleuca alternifolia*, *Eucalyptus globulus*, *Mentha piperita*, and *Thymus vulgaris,* were tested against ESBL-producing *E. coli*, *K. pneumoniae*, metallo-beta-lactamase (MBL)-producing *P. aeruginosa*, and carbapenemase (KPC)-producing *K. pneumoniae* [146]. Results showed that *M. alternifolia* and *T. vulgaris* EOs exhibited the best antibacterial activity, with MICs ranging from 0.5 to 16 µg/mL [146]. *M. alternifolia* EOs was the most effective, outperforming reference antibiotics [146].

While this review demonstrates the promising antibacterial activity of various EOs against numerous MDR pathogens, it is important to note that not all WHO priority pathogens have been adequately tested. Within the scope of this review, significant gaps remain in the literature regarding EOs efficacy against certain critical pathogens, including. *Mycobacterium tuberculosis* (rifampicin-resistant), *Neisseria gonorrhoeae* (cephalosporin/fluoroquinolone-resistant), vancomycin-resistant *Enterococcus faecium*, and Shigella species.

### 3.3. Methods Adopted for Testing the Antibacterial Activity of Plant Essential Oils

A rigorous evaluation of EOs’ antibacterial activity requires a diverse methodological toolkit, encompassing traditional antimicrobial screening and MIC determination, as well as advanced structural, molecular, and computational approaches. The selection of appropriate methods depends on the research question, whether it involves conducting preliminary activity screening, investigating mechanisms of action, assessing biofilm disruption, or evaluating the clinical translation potential. However, the lack of standardized protocols across studies remains a critical barrier, limiting reproducibility, cross-study comparability, and clinical integration of findings. This section reviews current methodologies employed in EO antibacterial research (Table 2), highlighting their applications, limitations, and best practices for generating robust and translatable data.

#### 3.3.1. Microbiological Techniques for Evaluating EOs Antimicrobial Activity

In vitro evaluation of EOs antimicrobial activity employs two primary methods: dilution methods (agar or liquid broth) [9,42,52,111,114,132,137,147,148,192,196] and diffusion methods (disk or well diffusion) [32,43,120,121,132,154,168,192,197,198]. Disk diffusion is a simple, cost-effective preliminary screening tool measuring inhibition zone, but it’s unsuitable for highly volatile compounds due to rapid evaporation [199].

While agar diffusion methods are useful as preliminary screening, they should be complemented with dilution-based assays for quantitative assessment [187]. To address EOs’ hydrophobicity without inhibiting microbial growth, solvents such as 0.15% agar or Tween 80 (0.1–2% *v*/*v*) are commonly employed [187].

For precise quantification, dilution methods, particularly broth microdilution assays [32,42,111,128,130,131,136,137,148,155,181,196,197,200], enable the determination of MIC and minimum effective concentrations (MEC) [139,201]. Reported EO MIC values range from 6.25 to 100 µL/mL [30,153], although variability arises from differences in EOs solubility and assay conditions [187].

To enhance reproducibility, standardized parameters are recommended: 6 mm disks loaded with 10 µL of EO for disk diffusion, and solvents such as DMSO or ethanol validated at low concentrations (≤1%) to avoid artifactual inhibition, particularly for fastidious strains [187]. All assays should be performed in triplicate, with MICs reported in mass-based units (mg/mL) for cross-study comparisons [187].

Volatilization assays combine broth microdilution with vapor-phase methods for rapid screening of volatile compounds [178,202]. Modified protocols using agar Petri dishes with central glass vials are required due to high volatility and hydrophobicity [203]. Bioautography combines chromatography with antimicrobial testing, overlaying separated EO components on chromatographic plates with microbial suspensions to identify active compounds [20,114,203]; however, it remains unsuitable for volatile compounds [204].

Time-kill assays [20,52,69,89,116,136,155,181,202] confirm bactericidal effects by plotting viable cell numbers over time, providing insights about time-dependent efficacy [205,206]. The bactericidal or bacteriostatic effect of the EOs can also be determined by analyzing the time to death (survival curve) in a nutrient medium, where the number of viable cells in the medium after EOs addition is plotted against time. Sometimes, a combination of various methods is used for EO bioassays [205,206].

Challenges in the aforementioned methods include discrepancies in data due to varying MIC standards, difficulty in detecting interactions between different antimicrobial agents, and limitations in assays for volatile compounds. Comprehensive reviews of antimicrobial susceptibility testing methods provide further details [179,186,205,206,207,208,209].

#### 3.3.2. Techniques for Studying Antimicrobial Mechanisms

Understanding how EOs exert their effects requires mechanistic investigation. Both conventional and advanced techniques are employed to elucidate EOs’ impacts on biofilm formation, membrane integrity, gene regulation, and cellular metabolism [188,209]. This section progresses from foundational methods to cutting-edge approaches that provide molecular-level insights.

##### Conventional Methods

Foundational techniques include microtiter plate assays [111,139,201,210] and micro-atmosphere methods [52] for assessing EOs’ effects on biofilms and microbial growth. Biofilm studies utilize surface coating with inhibitors, biofilm inhibition assays [148,210,211,212], and light microscopy [209,210]. Virulence factor expression and activity are examined through skim milk agar [143], azocasein assays [148], and swarming motility assays [148,149]. Molecular techniques, including RNA isolation [111,149] and the ethidium bromide cartwheel (EtBr-CW) method [96,197,211], investigate gene regulation related to AMR and biofilm formation. Analytical methods such as bacterial surface charge measurement, checkerboard assay [20,43,52,96,114,115,128,130,142,147,148,153,156,200,213], PCR [130], and UV–visible spectra [169] characterize physicochemical properties and antimicrobial interactions.

##### Advanced Methods

Building on these foundational approaches, advanced techniques provide deeper mechanistic insights at the molecular and ultrastructural levels. For instance, biofilm studies employ XTT viability assay [188], scanning electron microscopy (SEM) [35,135,139,148,149,150,157,168,181,201], QS inhibition bioassays using *C. violaceum* CV026 [149], confocal laser scanning microscopy (CLSM) [148,149], and EPS inhibition assays [148]. Molecular techniques, including real-time quantitative PCR (RT-qPCR) [96,139,197,201,211] and proteomic expression validation through qRT-PCR analysis [52,89], offer high sensitivity in studying gene and protein expression changes in response to EOs treatment. Analytical methods include bioluminescence assays [10,85], resazurin microplate assay [52,115,148], X-ray diffraction (XRD) [158], Fourier transform infrared spectroscopy (FTIR) [158], and transmission electron microscopy (TEM) [155,158] for detailed structural and functional characterization. The MTT viability assay, based on the reduction of the yellow tetrazolium dye 3-(4,5-dimethylthiazol-2-yl)-2,5-diphenyltetrazolium bromide (MTT), evaluates cytotoxicity of EOs for therapeutic safety assessment [139,150,157].

These comprehensive techniques enable a deeper understanding of Eos’ mechanisms, efficacy, and applications in combating microbial infections and resistance, though limitations include high costs and implementation complexity.

#### 3.3.3. Specialized Techniques and Emerging Techniques

Beyond conventional mechanistic approaches, specialized and emerging techniques offer unprecedented resolution and novel perspectives on EO–bacteria interactions. These cutting-edge methods, including single-cell analysis, computational modeling, microfluidic platforms, and multi-omics integration, are transforming our understanding of EOs’ antibacterial mechanisms and accelerating translational research.

##### Single-Cell Analysis Techniques

Single-cell analysis techniques provide high-resolution insights into EOs’ antibacterial effects. Flow cytometry accurately and rapidly assesses bacterial viability [130,192]. It detects viable but non-culturable (VBNC) subpopulations missed by traditional methods, yet requires improved dual staining systems [159,160]. Raman spectroscopy effectively quantifies major EOs compounds and detects adulterations when combined with chemometrics [170,193], but it struggles with low-content substances and volatile EO nature, requiring specialized setups and comprehensive spectral databases [170,193].

##### Computational Approaches

Computational methods, including molecular docking, dynamics simulations, and machine learning, enable detailed analysis of EOs-bacterial target interactions and predict antibacterial activity based on chemical composition. These techniques combine in vitro and in vivo studies, provide novel insights, enhanced accuracy, and high-throughput screening potential. However, implementation requires specialized equipment, expertise, and multidisciplinary collaboration [100,158,161,167].

##### Microfluidics and Lab-on-a-Chip Devices

Microfluidics and lab-on-a-chip (LOC) devices offer powerful tools for studying EOs’ antibacterial and antibiofilm activities [162,164,214]. These platforms enable high-throughput screening, real-time monitoring, and precise spatiotemporal control with enhanced sensitivity and rapid mass and heat transfer. Their high surface area-to-volume ratio intensifies antibacterial activity, achieving near-complete bacterial inhibition within minutes compared with hours required for traditional methods. Additionally, they require minimal reagents and samples, reducing costs and environmental impact. Researchers have utilized microfluidic chips to evaluate EOs nanoemulsions through cytoplasmic constituent release measurement and computational fluid dynamics (CFD) modeling [16,162,164], facilitating differential GNB and GPB effect studies via desorption electrospray ionization (DESI) and atomic force microscopy (AFM) [215]. Implementation requires specialized setups and interdisciplinary collaboration [162,164,214,215].

##### Omics Approaches

Omics technologies enable comprehensive, multi-component analysis and systems-level approaches to study complex molecular EOs–target interactions, overcoming conventional method limitations [52,216,217]. However, a critical challenge remains in pinpointing which specific molecules are indispensable for observed bioactivity, both in vitro and in vivo. This ambiguity complicates quality control and validation of EO batches for therapeutic applications, as even minor compositional variations, driven by plant genotype, environment, or extraction methods, can alter efficacy. Reductionist single-active molecule approaches exclude minor component contributions and synergistic interactions, while testing based on abundant molecules often fails to capture original phytocomplex bioactivity [216,217]. Integrating multi-omics data with bioinformatics and in silico approaches identifies multi-component, multi-target interactions, addressing the limitations of conventional bioactivity-guided fractionation [217].

Genomic data can help identify genes and regulatory elements involved in the biosynthetic pathways of EOs components, providing insights into their mode of action [218,219,220]. When integrated with metabolomics, which analyzes complete EO metabolite profiles using GC-MS and NMR, and advanced analytical techniques like flow-modulated comprehensive two-dimensional gas chromatography coupled with mass spectrometry (FM-GC × GC-MS) and chemometrics provides improved chemical analysis and metabolic profiling, enabling comprehensive characterization of plant metabolites [173].

Transcriptomics examines gene expression patterns in EO biosynthesis and metabolic pathways, studying the complete RNA transcripts produced by genomes under specific conditions using microarrays and RNA sequencing (RNA-Seq) [220,221]. Lai (2020) used microarray transcriptomic analysis to elucidate piperacillin and *Lavandula angustifolia* EOs synergistic activity against MDR *E. coli* K-12, identifying 90 differentially expressed genes with biochemical pathway analysis showing upregulation of genes in numerous biological processes and up/down-regulation of microbial processes [174].

Metabolomics analyzes complete EO metabolite profiles using GC-MS and NMR, providing global metabolic composition overviews and identifying biomarkers or biosynthetic pathways [189,194]. For instance, metabolomic analysis has revealed how EOs disrupt bacterial quorum sensing by affecting tryptophan metabolism pathways [56], while integrated metabolomic–transcriptomic studies have shown that sublethal EO exposure triggers bacterial adaptive responses involving aminoacyl-tRNA biosynthesis and alterations in 47 metabolites including lipids, amino acids, and nucleotide-related compounds [189,219].

Proteomics examines complete protein sets to reveal enzymes and regulatory proteins involved in EO biosynthesis and antibacterial action, complementing transcriptomic and metabolomic data by linking gene expression to functional outcomes [52,56,195]. Proteomic studies have confirmed that EOs induce oxidative stress and membrane disruption in resistant pathogens, with qRT-PCR validation demonstrating concordance between protein abundance and gene expression profiles [89]. Comparative proteomic analysis has also enabled identification of the most potent antibacterial constituents within complex EO mixtures; for instance, thymol was identified as more effective than carvacrol in Origanum vulgare EO through its interference with protein regulation and DNA synthesis at sub-lethal concentrations [195].

The integration of omics data highlights EO bioactivity as an emergent property of complex mixtures. However, this very complexity challenges the identification of critical active constituents and the reproducibility of therapeutic effects. Future work must bridge omics insights with bioassay-guided fractionation to validate consistent markers for batch-to-batch quality control, ensuring reliability in EO-based therapies.

## 4. The Challenges for the Development of Essential Oils as Therapeutics

Despite the rapid progress and mounting interest in EOs for their promising antimicrobial properties, multiple complex technical, regulatory, and safety hurdles must be considered prior to their successful implementation in clinical settings. Issues of consistency and standardization, environmental sustainability, pharmacological and stability complexities, regulatory requirements, and clinical translation represent some of these challenges. Understanding these limitations is crucial for researchers, healthcare providers, and regulatory bodies to develop effective strategies to leverage the therapeutic potential of EOs while also ensuring their safety and efficacy in clinical applications. A summary of some of these challenges is presented below.

### 4.1. Variability of Essential Oils Yields and Bioactivity

A significant limitation in EO research resides in the substantial variability in EO yields and bioactivity, which affects data interpretation and cross-study comparability of antimicrobial efficacy data. This compositional heterogeneity is particularly evident when examining EOs bearing identical botanical nomenclature across different studies. For example, Ceylon cinnamon (*Cinnamomum verum*) demonstrated compositional differences across multiple studies, where cinnamaldehyde concentrations ranged from 64.49% to 72.81%, while eugenol concentrations varied dramatically from 6.57% to 77% [111,148,149,150,152]. Similarly, peppermint (*Mentha piperita*) exhibited variability in the concentrations of menthol from 43.66% to 68%, and menthone from 8.36% to 24.43% [89,128,155,171]. The interconnected factors affecting EOs yields and bioactivity are detailed below.

#### 4.1.1. Endogenous and Exogenous Factors

Plant EO composition and bioactivity are affected by endogenous factors (genetic makeup, chemotype, developmental stage, target organ/tissue) and exogenous factors (biotic activities, abiotic stress, extraction methods, post-harvest handling/storage) [39,172,183,222]. Distinct chemotypes within species exhibit different chemical profiles without morphological differences, substantially modifying EO activity through environmental factors like hybridization, soil quality, climate, altitude, cultural practices, and genetic factors [223].

#### 4.1.2. Plant Age and Development

The metabolic pathway activity, environmental conditions, and genetic factors affect the relationship between plant age and essential oil production [223], contributing to distinct patterns across plant species, developmental stages, and exogenous conditions [223]. While some studies reported a decline in EOs synthesis with increasing plant age [175,176,190,224,225,226], potentially attributable to declining metabolic pathways, others have documented optimal yields at specific ages (6 and 15 months) [175,225], or enhanced chemical composition in mature compared with younger ones [191].

#### 4.1.3. Plant Part Variability

EOs composition, yield, and quality vary significantly across plant parts (flowers, seeds, roots, stems, leaves) depending on genotype, chemotype, developmental stage, season, geographic locations, and harvest timing [173,190,222,227,228,229]. Rathore and Colleagues (2023) demonstrated that genotype and cultivar affect the predominant EOs constituent amounts within *Cymbopogon winterianus* across Western Himalayan regions [229]. Multiple studies confirm similar findings [15,115,161,173,182,185,230,231,232,233,234,235,236].

#### 4.1.4. Geographic and Environmental Influences

The geographic origin of plant EOs has a predominant effect on its composition and yields [39,237]. Al-Kharousi and Colleagues (2023) found that the differences in the composition of frankincense (*Boswellia sacra*) oil were primarily related to extraction method, harvest time, and tree incision number rather than climate/geographic location [231]. Facanali and Colleagues (2020) [173] investigated three *Varronia curassavica* Jacq. Genotypes, finding spring and summer as optimal harvest seasons with the highest active ingredient yields and higher α-humulene concentrations in the VC2 genotype [173,238].

Limonene composition in Citrus lemon oil varies by location [233]. Japanese lemon leaf oil contained geranial, limonene, and neral, while Egyptian lemon leaf oil included predominantly caryophyllene, linalool, nerol, and limonene [233]. Italian, Turkish, and Chinese lemon leaf oils contained limonene, β-pinene, and geranial, while Benin oils primarily consisted of limonene, β-pinene, and citronellal [234]. Dalli (2021) showed that *Nigella sativa* seed EOs yields and chemical composition differed between Morocco, Saudi Arabia, Syria, and France [237].

#### 4.1.5. Seasonal Variations

Seasonal variations significantly impact EO chemical profiles and bioactivities, with maturity stages interlinked to seasonal progression, creating disparate phenological patterns within species due to unique local ecological responses [223,230]. Motsa (2006) observed similarities between *Nectandra megapotamica* and *N. Nectandra lanceolata* EOs during spring/autumn but differences in summer/winter [236] *N. megapotamica* winter/spring EOs showed the highest *E. coli* inhibition due to monoterpenes (α-pinene, β-pinene, myrcene, limonene) [239], while N. Lanceolata summer/autumn EOs exhibited lower S. aureus MIC due to sesquiterpene hydrocarbons [236].

Machado and Colleagues (2014) demonstrated seasonal fluctuations in *Lippia alba* leaf EO antimicrobial potency due to phytochemical profile changes related to environmental conditions, with varying effectiveness against different species: *S. aureus* (December-February), *Listeria monocytogenes* (June-August), and *L. innocua* (December-August) [232].

#### 4.1.6. Environmental Factors

**Figure 1:**
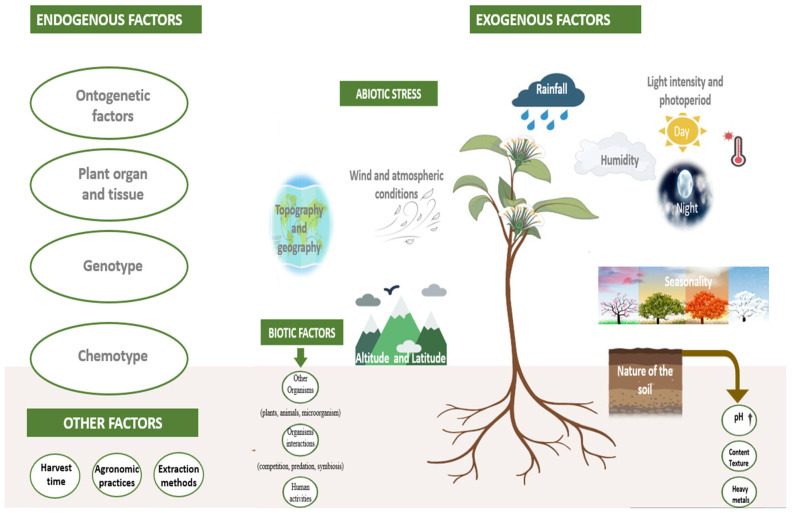
Factors affecting the variability in EOs’ composition and yields.

Environmental factors, including rainfall, temperature, humidity, life cycle stage, sunlight exposure, night light, wind patterns, soil characteristics, and heavy metal content, profoundly influence plant growth and development, consequently affecting EOs’ quantity and quality [223] (Figure 1).

#### 4.1.7. Extraction Methods

The selection of the extraction method impacts stereochemical and physicochemical properties, chemical composition, and biological activity [15,41,172,240]. EOs from identical species extracted using different techniques contain distinct constituents that affect biological activity [15,172,185,235]. Notably, heat-based extraction methods may create artifacts from chemical precursors not naturally present in the plant, potentially altering the final EOs composition [172].

Numerous extraction techniques, ranging from conventional and widely employed methods to emerging eco-friendly approaches, are outlined in Appendix A.1.

The conventional methods of EOs extraction remain the most commonly used methods at industrial scales [241,242,243]. However, their inherent limitations, including high energy consumption, adverse effects on heat-sensitive compounds, low extraction efficiency, potential loss of volatile compounds, and the formation of toxic solvent residues in EOs [241], have driven a shift toward greener extraction techniques [241,242,243]. Advanced methods offer several advantages over conventional approaches [241,242,243], including enhanced quality and purity of EOs. However, they generally require higher resource allocation, careful optimization, and adjustment of parameters such as temperature, enzyme types, and concentrations depending on the plant material [240,243]. These techniques are more complex than conventional methods, and newer technologies necessitate more research and development [243]. A comparison between conventional and advanced methods is summarized in Appendix A.2.

End-product quality control of EOs consists of using analytical techniques designed to cope with volatile compounds. The most used method is gas chromatography (GC) coupled to either a mass spectrometer (MS) or a flame ionization detector (FID) [244]. More recent techniques have included GC-IMS (Ion Mobility Spectrometry) as a viable alternative for end-product quality control, indicating that this newer technique can discriminate between the geographic origins of oils [245]. In-process controls are needed to ensure that the extraction process of the EOs complied with the process validated for the manufacture of the EOs as a therapeutic developed according to the Quality Guidelines of the International Council for Harmonisation of Technical Requirements for Pharmaceuticals for Human Use [246].

### 4.2. Safety Concerns

According to the U.S. Food and Drug Administration (FDA), numerous EOs are generally recognized as safe (GRAS) [247], meaning consumer exposure do not “exceed the amount reasonably required to achieve intended physical, nutritional, or other technical effects in food” [248]. Consequently, this classification allows including EOs in cosmetics, food, and feed applications but precludes their use as therapeutic agents. However, at the concentrations required for therapeutic application, some EOs exhibit toxic effects, including respiratory problems, acute toxicity, reproductive toxicity, and organ toxicity, even at very low concentrations [249].

Studies have highlighted potential adverse effects including skin sensitization, contact dermatitis, neurological toxicity, and endocrine disruption, emphasizing safety considerations particularly during pregnancy and lactation, as some EOs components may cross the placental barrier and pose fetal risks [249,250]. Specific EOs have exhibited teratogenic effects (*Eucalyptus staigeriana* [163], *Thymus* spp. [165], *Salvia lavandulifolia* [251]), embryotoxic effects (*Curcuma zedoria* [252]), neurotoxic effects (*Artemisia vulgaris* [253]), and nephropathic, carcinogenic, and genotoxic effects (*Mentha piperita* [254]). Additionally, *Salvia officinalis*, *Hyssopus officinalis*, lavender and tea tree oils exhibited neurological toxicity and endocrine-disrupting effects [255]. Some studies have generated contradictory findings regarding EO toxicity in vitro and in vivo [249]. Some EOs exhibited toxic properties even at very low concentrations, potentially due to variations in their chemical composition [249,254].

Therefore, before gaining drug approval, EOs must undergo extensive toxicological assessments according to safety guidelines established by the International Council for Harmonisation of Technical Requirements for Pharmaceuticals for Human Use [246], which represent minimum requirements by major regulatory agencies worldwide [246]. Comprehensive EO toxicity assessment determines safe and toxic concentration ranges, exposure time, toxicity mechanisms, and specific constituents responsible for these effects [253,256]. Methods involving in vitro cytotoxicity assays, in vivo models using non-mammalian organisms (e.g., *Caenorhabditis elegans*), hen’s egg test, gene expression analysis, mucous membrane irritation testing, and chemical profiling are essential for ensuring safe and effective EOs utilization in therapeutic settings [253]. These approaches aim to provide an understanding of EO toxicity while reducing reliance on traditional animal models [253].

### 4.3. Supply and Environmental Concerns

Securing the continuity of supply is crucial before a drug gains marketing authorization. The consistency of the supply often leads to the often-overlooked environmental issue during phytochemical drug development about whether sufficient plant material will be available to produce the drug annually. For example, before synthetic Taxol manufacturing, the raw material for the drug was harvested from the bark of plants, and, for the Taxol clinical trial alone, 12,000 trees were harvested to obtain sufficient bark to extract the drug compound [257]. A further issue complicating the supply of EOs for clinical use is the correct selection of the plant species used for the initial identification and subsequent clinical evaluation of the therapeutic EOs [166]. Despite not having occurred with an EO, this problem was raised in 1997 when plantain, a herbal remedy and culinary ingredient, was contaminated with *Digitalis lanata*, leading to significant, unexpected cardiac side effect [258].

### 4.4. Regulatory Landscape

The challenges of obtaining regulatory approval for a therapeutic agent developed from a complex plant extract rather than a single molecule is immense. This issue often revolves around the challenge of identifying single or multiple active molecules in the chromatogram of the EOs for development into a therapeutic agent. The same concern applies to identifying potentially toxic compounds that may be present in the product [259].

The EO industry is subject to a complex regulatory landscape covering quality standards, ethical sourcing, labeling, and international trade [31]. Regulation and standardization of essential oil products vary globally [34], leading to different quality control and labeling. Numerous countries have their regulatory bodies, while others follow the WHO, Food and Agriculture Organization (FAO), the FAO/WHO Codex Alimentarius Commission (CAC), and the International Organization for Standardization (ISO) for the use of EOs [260]. These organizations work together to develop strategies and implement regulations for the safe use of EOs globally. The WHO provides a digital platform addressing the safety of plant materials, publishes guidelines on good manufacturing practices for herbal medicines, and issues scientific information on the safety and quality of EOs [260]. The WHO and FAO also established committees to evaluate the safety levels of food additives, including EOs. The national entities in different countries regulate EOs, and their authentication can be difficult, requiring various techniques, including chiral gas chromatography, isotope-ratio mass spectrometry, nuclear magnetic resonance (NMR), thin-layer chromatography, vibrational spectroscopy, multidimensional chromatography, high-performance liquid chromatography, headspace chromatography, and chemometrics-metabolomics [145]. All these aspects should be incorporated into the different guidelines [261,262] that emerged based on specific cultivation and processing methods. Examples include the WHO Guidelines on Good Agricultural and Collection Practices (GACP) for Medicinal Plants [263], WHO guidelines for sampling of pharmaceutical products and related materials [264], and the European Medicines Agency (EMA) Guideline on Quality of Herbal Medicinal Products (CPMP/QWP/2819/00)[265].

Given the current progress that has been made in the field of regulations for EOs, there remains a pressing need to reassess the EOs mechanism of action and support rigorous studies to explore their full potential for human health within the framework of integrative health approaches [266].

### 4.5. Pharmacokinetic and Pharmacodynamic Properties

Due to the complexity of EOs’ chemical composition, volatility, and susceptibility to degradation, it is crucial to have a better understanding of their bioavailability and kinetics [267] to allow the discovery and identification of EO candidates for clinical practice [17]. The EOs’ pharmacodynamic properties were studied in vitro. Their target organs and safety profiles lack confirmation in vivo due to various degradative pathways and enzymes [32]. Therefore, further studies on absorption, distribution, metabolism, and excretion are necessary to establish the relationship between in vitro and in vivo investigations [268]. Adopting multimodal approaches is essential to comprehensively examine the intricate interplay between chemical profiles of EOs and their respective metabolites, mobilome, resistance, and metabolism in cohabiting bacteria, and this appears to be particularly crucial in ecological settings where community-driven resistance selection occurs [13]. The complexity and variability of EOs compositions make it difficult, if not impossible, to explore their bioavailability in vivo.

Plant extracts, including components of EOs, have been shown to affect drug metabolism by affecting the activity of cytochrome enzymes [144] or drug transporters [269], and may affect the activity of other drugs concomitantly taken by the patient. EOs are volatile substances and prone to oxidation and enzymatic breakdown. This issue may necessitate encapsulation to be delivered successfully to the body without losing activity, adding to the complexity of the drug development process and cost [270].

Another challenge is the reluctance to develop a biofilm-dismantling product without biocidal activity due to concerns that disrupting the biofilm could lead to bacteremia [271].

### 4.6. Drug Interactions and Delivery

EO components can affect drug metabolism by influencing cytochrome enzyme activity and drug transporters, potentially impacting the efficacy of other medications [31]. The volatile nature of EOs and susceptibility to oxidation and enzymatic breakdown may require advanced delivery methods, such as encapsulation, adding to development costs and challenges [270]. Understanding the potential interactions of EOs with medications is crucial, as it can affect their effectiveness and lead to adverse effects. This is especially important given that the mechanism of action of EOs as a multicomponent mixture (more than one constituent substances (MOCS), including hundreds of individual compounds), has shown diversity and therapeutic advantages with fewer side effects compared to using a single compound [31].

### 4.7. Co-Administration Challenges

The multi-component nature of EOs complicates trial design, especially when testing their synergistic effect in combination with standard antibiotics. This issue is problematic in Phase 3 clinical trials, as the co-administration of drugs usually requires a superior primary endpoint. For example, van Vuuren and Colleagues (2009) [272] explored the interactions of EOs, including *Melaleuca alternifolia*, *Thymus vulgaris*, *Mentha piperita*, and *Rosmarinus officinalis* with ciprofloxacin. When combined with ciprofloxacin against *S. aureus*, the EOs exhibited primarily antagonistic effects [272]. However, against *K. pneumoniae,* the combinations produced varied outcomes, with isobolograms indicating antagonistic, synergistic, and additive interactions depending on the ratio used [272]. The *R. officinalis*-ciprofloxacin combination showed the most favorable synergistic effect against *K. pneumoniae* [272]. This variability in interaction suggests that the effectiveness of EOs combined with antibiotics is highly dependent on their specific ratios, and caution is advised when using such combinations to avoid reducing therapeutic efficacy [272].

### 4.8. Research Translation in Clinical Care

Clinical trials are required to confirm the efficacy and safety of MOCS in treating infectious diseases under various health conditions, including cancer, obesity, diabetes, and cardiovascular diseases, due to the complexity of EOs and taking into consideration the potential synergism with conventional antibiotics [31,273].

Other relevant studies include evaluating bacterial resistance and tolerance to EOs, as well as determining whether their use as adjunctive therapy can prevent cross-resistance to antibiotics and antagonistic effects [8,9].

Another challenge in translating EO research lies in the limited systematic comparisons between laboratory reference strains and clinical isolates. While direct head-to-head studies remain scarce, this gap represents a fundamental barrier to predicting clinical efficacy from laboratory data, requiring more rigorous comparative research to guide therapeutic development.

Beyond laboratory or clinical settings, studying the adherence to EO-based treatments, integration of interventions into healthcare practices, and public acceptance of these interventions need evidence-based practices and extensive research.

## 5. Understanding Essential Oils Resistance Development

The potential for bacteria to develop resistance against EOs mandated extensive investigations into their complex mechanisms of action and adaptive responses. While numerous studies reported no significant development of direct genetic resistance, likely due to the complex compositions and multi-targeted mechanisms of EOs [10,17,180,274], others have observed the emergence of resistant variants or decreased susceptibility [274,275,276,277]. Notably, the emergence of resistant variants appears strain-specific, with some bacterial species exhibiting resistance or tolerance development against certain EOs or their components, while others remain unaffected [274,276,277]. There are several possible mechanisms through which AMR could develop. One of these mechanisms is the overexpression of efflux pumps. Increased efflux pump activity can lead to reduced intracellular concentrations of EOs, thereby limiting their antimicrobial activity. Moken and Colleagues (1997) studied *E. coli* isolates with resistance against pine EOs, potentially mediated by mutations of the multiple antibiotic resistance (*mar*) locus [278]. This region controls the expression of multiple resistance mechanisms, including efflux pumps [278]. It is also associated with target modification and decreased membrane permeability, potentially due to the downregulation of outer membrane protein F (OmpF) and other membrane proteins [279].

Another gene, *soxS*, was activated as well, and it induces the expression of ROS genes, which serve as an antioxidant defense system. This mechanism allows bacteria to overcome oxidative stress induced by EOs [278,280]. Studies showed that, while direct bacterial resistance is limited, sublethal EOs exposure can induce cross-resistance or decreased susceptibility to antimicrobial agents, heat, and oxidative stress [220,275,277]. This phenomenon is due to phenotypic adaptations, such as altered membrane compositions, efflux pump activity, metabolic pathways, and gene expression profiles [177,220]. Pagan and Colleagues (2024) observed a lack of uniform genotypic or phenotypic patterns in *S. Typhimurium* lineages exposed to carvacrol, underscoring the complexity and unpredictability of adaptive responses [277]. While the overall risk of resistance development against EOs is lower than conventional antibiotics, continuous monitoring and responsible use are imperative, particularly in real-world scenarios where sublethal EOs stress may contribute to cross-resistance emergence [220]. Nonetheless, the rate of AMR development toward EOs may be lower than conventional antimicrobials. Becerril and Colleagues (2012) investigated the emergence of AMR against cinnamon oil by *E. coli*, *P. aeruginosa*, *S. marcensces*, *Morganella morganii*, and *P. mirabilis* [274]. After 50 passages in the presence of cinnamon EOs, *E. coli* strains failed to grow, and none of the other bacterial strains developed resistance toward cinnamon EOs [274]. Furthermore, isolates treated with cinnamon oil, unlike untreated ones, could not develop resistance against different antimicrobial classes, including β-lactams, aminoglycosides, tetracyclines, quinolones, and chloramphenicol [274]. That is probably due to the complex composition and highly diverse nature of EOs and their multiple-target approach. Thus, bacteria would need to simultaneously evolve multiple resistance mechanisms against numerous processes to become fully resistant to EOs [17]. Additionally, EOs often exhibit a synergistic effect among their constituents or when combined with other EOs or antibiotics, which can reduce selective pressure and hinder the development of AMR [12]. In addition, EOs have reportedly overcome common resistance mechanisms that are usually effective against traditional antibiotics, such as inhibition of biofilm formation and efflux pumps [37,281].

## 6. Potential Advantages of Using Essential Oils in the Fight Against AMR

### 6.1. The Multi-Target Mechanisms of Plant EOs Against Antibiotic Resistant Clinical Isolates

EOs demonstrate extensive antimicrobial activity against drug-resistant bacteria, with significant contributions from multiple plant families. The Lamiaceae family emerges as a dominant source, with oregano (*Origanum vulgare*) [43,70,129,130,195], thyme (*Thymus vulgaris*) [44,52,96,139,140,162], lavender (*Lavandula angustifolia*) [153,181,210], and peppermint (*Mentha × piperita*) [41,128,155,196,200] exhibiting broad-spectrum activity. The Myrtaceae family, represented by the tea tree (*Melaleuca alternifolia*) [60,136,137,282] and clove (*Syzygium aromaticum*) [117,118,123,197], shows potent efficacy, particularly against respiratory pathogens, while the Lauraceae family’s cinnamon EOs demonstrates exceptional activity against pan-drug-resistant (PDR) strains [112]. Apiaceae (*Coriandrum sativum*) [122,213,283], Geraniaceae (*Pelargonium graveolens*) [40,284], and Poaceae (*Cymbopogon flexuosus*) [41,112,127,189] families show additional significant antibacterial effect. Plant EOs exert multifaceted mechanisms of action against resistant bacteria through five primary pathways: direct membrane disruption with precisely documented MIC values ranging from 0.0562 to 512 μg/mL [43,112], anti-biofilm activity achieving 48–90% reduction in biofilm formation [213], QS inhibition that disrupts bacterial communication networks [69,85,152,201,282], efflux pump inhibition that restores antibiotic susceptibility [142,177,208,278], and genetic modulation of resistance mechanisms [70,148]. This mechanistic versatility is exemplified by cinnamon oil’s remarkable dual action against colistin-resistant strains, achieving both direct antibacterial effects and molecular-level intervention, demonstrated by 20–35-fold reductions in mcr-1 gene expression in both colistin-resistant *P. mirabilis* and *E. coli* [113]. Structural analyses reveal critical component-specific activities: cinnamaldehyde demonstrates superior antimicrobial action compared to eugenol against *P. aeruginosa* (MIC: 0.00002–0.03 μL/mL) [111], while terpinene-4-ol, a key tea tree oil component, shows significant broad-spectrum activity (MIC: 0.048–1.52 mg/mL) [282]. The molecular basis of bacterial resistance modulation is particularly noteworthy: various oils demonstrate anti-efflux-pump activity, with menthol-imipenem combinations achieving a 16-fold reduction in imipenem MIC against resistant strains while simultaneously downregulating resistance-associated genes, including *clbB* and *mcr*-1 [42]. Against specific pathogens, the activity spectrum is comprehensive: cinnamon oil shows remarkable efficacy against PDR *P. aeruginosa* (MIC: 0.0562–0.225 µg/mL) through both direct action and *mcr*-1 gene downregulation, tea tree oil demonstrates consistent activity against MRSA (MIC: 0.048–3.125 mg/mL) [282], thyme oil specifically targets *Streptococcus pneumoniae* (MIC: 0.625–1.25 μL/mL) [96], and oregano oil exhibits exceptional activity against carbapenem-resistant Enterobacteriaceae (MIC: 0.015% *v*/*v*) [129]. Synergistic interactions with conventional antibiotics show particular promise across multiple combinations: lavender oil with meropenem against carbapenem-resistant KPC-producing *K. pneumoniae* (MIC reduction from 10% to 0.63% *v*/*v*) [52], rosemary and geranium oils with colistin against extensively drug-resistant (XDR) *A. baumannii* (2–32-fold MIC reduction) [285], and various combinations with β-lactams and aminoglycosides. Clinical applications demonstrate targeted efficacy: tea tree, immortelle and eucalyptus oils against respiratory tract isolates [32,286], oregano, cinnamon and rosemary EOs against uropathogens [129,134,135,197], and geranium, black cumin and tea tree against wound pathogens [120,282,284], with potent anti-biofilm activity from cinnamon and peppermint oils (optimal at 30 μL/100 μL) [121]. An important aspect highlighted in the research is the ability of certain EOs to modulate bacterial resistance mechanisms, with several oils demonstrating anti-efflux pump activity and the ability to downregulate resistance-associated genes [42,184]. The potential of EOs in preventing biofilm formation is a crucial factor in bacterial persistence and AMR, with EOs such as cinnamon and peppermint EOs showing significant anti-biofilm activity at sub-inhibitory concentrations, suggesting potential applications in preventing bacterial colonization without promoting resistance development [121].

### 6.2. Synergistic Effect of Plant EOs and Conventional Antibiotics

Plant EOs demonstrated potentiating action on conventional antibiotics such as penicillins, cephalosporins, quinolones, chloramphenicol, and sulfamethoxazole-trimethoprim [12,18,19,22,23,287]. They can also re-sensitize MDR [12]. The EOs effectiveness was reported both alone and in synergy with antibiotics against resistant GPB and GNB, including MRSA, ESBL-producing *E. coli*, MDR *A. baumannii* [12], and carbapenemase-producing *K. pneumoniae* [52]. Knezevic and Colleagues (2016) demonstrated that *Eucalyptus camaldulensis* EOs in combination with ciprofloxacin, gentamicin, and polymyxin B exhibited MICs ranging from 0.5 to 2 μL/mL [20]. The combined application of EOs and conventional antibiotics significantly enhanced antibacterial efficacy and even resensitized MDR *A. baumannii* strains, particularly with polymyxin B, rapidly reducing bacterial counts [20]. Soliman and Colleagues (2017) [287] assessed the antimicrobial potential of Lawsone and Calli EOs against MDR pathogens. Lawsone EOs showed significant activity at 200–300 µg/mL, while Calli EOs were effective at 180–200 µg/mL against MDR bacteria [287]. Lawsone in combination with Calli EOs enhanced antimicrobial efficacy by at least three-fold, achieving ≥90% inhibition of all tested strains [287].

Dhara and Tripathi (2020) evaluated cinnamaldehyde, both alone and with cefotaxime and ciprofloxacin, against ESBL-producing and quinolone-resistant Enterobacteriaceae [18]. MICs were 7.34 µg/mL for E. coli and 0.91 µg/mL for *K. pneumoniae*. Synergistic interactions were found in 75% of *E. coli*, 60.6% of *K. pneumoniae* with cefotaxime, and 39.6% and 42.4% with ciprofloxacin, leading to a 2 to 1024-fold reduction in MICs [18]. Cinnamaldehyde also significantly altered bacterial morphology and gene expression related to porins, efflux pumps, and antibiotic resistance [19]. In another study, the same authors showed that Eugenol combined with cefotaxime and ciprofloxacin demonstrated substantial synergism (FICI: 0.08–0.5), reducing MIC values by 2- to 1024-fold and demonstrating a dose reduction index (17- to 165,030-fold) [18]. Eugenol alone or in combination disrupted bacterial cell structure, downregulated efflux pumps, overexpressed porins, and inhibited β-lactamase genes, thus reversing AMR [18].

Köse (2022) [23] demonstrated the synergistic effects of carvacrol combined with meropenem against carbapenem-resistant *K. pneumoniae* (CRKP) strains. MICs for both carvacrol and meropenem ranged from 32 to 128 µg/mL [23]. These findings highlight the complex and promising role of EOs-antibiotic combinations in addressing MDR [12,18,20,21,23,287].

However, inconsistencies in methods and interpretation criteria across studies warrant the need for further comprehensive research to understand the interactions and mechanisms of action and optimize these combinations for clinical use [22].

### 6.3. Nanoencapsulation of Plant Essential Oils

EOs nanoencapsulation is a promising strategy to enhance antimicrobial activity, particularly against antibiotic-resistant bacteria [17,256,288,289,290,291]. This technology offers several advantages over unencapsulated EOs, including bioactive protection from external environment [290,291,292,293,294], prevention of thermal oxidation reactions [17,239], lower cytotoxicity [17,239,294,295], and improved stability, solubility, bioavailability, and penetration ability [292,296].

Nanostructured delivery systems (NDSs) are molecularly composed of different biomaterials and processed in various forms to interact specifically with targets. Various nanocarriers have been explored for EOs encapsulation, including eco-friendly and biodegradable polymeric and lipidic nanodelivery systems such as chitosan-based systems (CHT) [289,291,297], liposomes [289,292,293], solid lipid nanoparticles, and nanoemulsions [289]. Other materials, such as cyclodextrins [296] and nanogels, have also shown potential [288,290]. The determination of EOs’ nanodelivery system, excipients, concentrations, and preparation method should be carefully planned to ensure successful encapsulation, long-term stability, high encapsulation efficiency, and therapeutic potency of bioactive EO-loaded nanocarriers [239]. The choice depends on the intended application, as size, shape, and component nature influence selection [17].

Synergistically combining EOs with potent antimicrobial nanoparticles (NPs) may potentiate collective antimicrobial efficacy through complementary mechanisms against diverse pathogens, appearing optimal for combating MDR microorganisms [17]. Nanocarriers facilitate controlled release [289,291,293], desirable shelf life [292], targeted delivery to specific sites, increased retention time and penetration into bacterial cells/biofilms [290,291], synergistic effects when combined with other antimicrobial agents, and minimized side effects [17,289,290,291].

Recent advances in biopolymeric nanoparticles research show that chitosan and zein-based nanosystems are highly effective in targeting antibiotic-resistant bacteria in both sessile and biofilm forms [291]. These biopolymer-based nanocarriers are convenient carriers due to their biocompatibility, low toxicity, and surface modification effect [291]. Nanoencapsulation enhances EOs’ antimicrobial and antibiofilm activity through increased retention time and penetration into bacterial cells/biofilms, synergistic effects with other antimicrobial agents, targeted delivery to infection sites, and prolonged therapeutic effects via sustained release [17,288,291]. Numerous studies demonstrate improved antibacterial and antibiofilm efficacy of nanoencapsulated EOs against *Pseudomonas* spp., *Staphylococcus* spp., and *E. coli* compared with free EOs [290,291,292,293].

However, multiple challenges remain to overcome for successful industrial adoption and clinical translation of nanoencapsulated EOs. Primary challenges include the need for large-scale, cost-effective production methods ensuring consistent quality and reproducibility [289]. Optimizing release kinetics, maintaining long-term stability, and investigating potential toxicity concerns require further research [239,291,298,299]. The complex structure and varying compositions of biofilms, depending on microbial strains, can make NP interaction prediction and effective delivery challenging [291]. Additionally, quantitative in vivo studies and specific dosage guidelines for EOs in different infections are currently lacking [289]. Nano-delivery system characteristics such as size, shape, surface functionalization, roughness, and charge significantly influence efficient EOs release and antimicrobial activity.

## 7. Conclusions and Future Directions

EOs represent a paradigm shift in antimicrobial therapy due to their multi-target mechanisms of action, which differ fundamentally from those of conventional single-molecule antibiotics. Their ability to simultaneously disrupt bacterial cell membranes, inhibit biofilm formation, interfere with QS, and modulate efflux pump activity positions them as particularly valuable weapons against AMR. The mechanistic diversity of EOs, exemplified by the efficacy of cinnamon, clove, oregano, and tea tree oils against WHO-priority pathogens such as MRSA, carbapenem-resistant *K. pneumoniae*, *A. baumannii*, and *P. aeruginosa*, demonstrates their potential to address urgent public health needs where conventional antibiotics have failed.

The synergistic potential of EOs with existing antibiotics emerges as one of their most clinically relevant attributes. This combinatorial approach not only enhances the efficacy of established antimicrobial agents but also provides a pathway to revive antibiotics that have lost effectiveness due to the development of resistance. The ability of EOs to inhibit β-lactamases, downregulate efflux pumps, and modulate bacterial gene expression provides multiple avenues for overcoming established resistance mechanisms.

However, the path to clinical implementation faces significant obstacles. The inherent variability in EO composition presents a fundamental challenge to standardization and reproducibility, indicating the need for robust quality control measures, standardized extraction protocols, and chemotype selection strategies. While nanoencapsulation technologies, including chitosan nanoparticles and liposomal formulations, offer promising solutions to enhance stability, bioavailability, and targeted delivery, the practical hurdles of scaling up production, managing costs, and navigating regulatory approval for nanoformulations remain substantial.

The current evidence base, while compelling, is heavily weighted toward in vitro and ex vivo studies, and well-designed clinical trials to validate efficacy and safety in human subjects are essential for future translation of EOs potential into clinical practice. The limited availability of in vivo models and clinical data represents a significant gap that needs to be bridged before EOs can be considered viable therapeutic alternatives. Additionally, while EOs are generally considered less prone to resistance development due to their multi-target nature, emerging evidence suggests that bacterial adaptation mechanisms, such as efflux pump upregulation, may still pose risks that warrant careful monitoring. Should EOs value be preserved as alternatives to conventional antibiotics, such adaptation and probable resistance emergence are potential venues for ongoing investigation.

The integration of artificial intelligence and machine learning approaches, as demonstrated by the successful application of artificial neural networks in predicting antimicrobial activity with >70% accuracy, offers a promising avenue for optimizing EO formulations and predicting therapeutic outcomes. These computational tools can help navigate the complex chemical variability of EOs and provide rapid, cost-effective screening methods for identifying optimal antimicrobial combinations.

Economic and feasibility considerations, while under-discussed in current literature, represent critical determinants of real-world adoption. The cost-effectiveness of EOs compared to conventional antibiotics, hurdles of their large-scale production, and market viability issues remain interesting to tackle.

In conclusion, essential oils present a compelling and scientifically sound approach to combating AMR through their unique multi-target mechanisms and synergistic potential with conventional antibiotics. However, successful clinical translation requires a coordinated effort to address standardization challenges, conduct rigorous clinical trials, develop advanced delivery systems, and optimize combination therapies. Research addressing these multifaceted aspects can help realize the full therapeutic potential of essential oils to tackle AMR, ultimately contributing to the preservation of antimicrobial efficacy for future generations.

## Figures and Tables

**Table 1:** Antibacterial activity of selected plant essential oils against resistant pathogens.

Essential Oils Proprietary Name (Species)/Family	Major Components	MIC	Targeted Bacteria	Isolation Source	Antimicrobial Mechanism	Ref.
Cinnamon and cinnamaldehyde/Lauraceae	Not available	Clinical isolates of *E. coli:* 32 μL per mL of cinnamon; MIC cinnamaldehyde ranges between 0.00002 to 0.03 μL/mL	*E. coli* carrying *pks* gene	Colon cancer patients, inflammatory bowel disease and healthy subjects	AB, ABF, AV	[111]
	Cinnamaldehyde (78.1%), benzyl alcohol (16.67%)	EOs MIC: 0.0562–0.225 μL/mL	Pan and Extensive Drug-Resistant *P. aeruginosa*	Burn wounds and urine samples	AB, AE	[112]
Cinnamon, Thyme, Eucalyptus/Lauraceae	cinnamaldehyde (E)—(40.91%), cinnamaldehyde dimethyl acetal (37.54%)	EOs MIC (Resistant strains): 4.88 to 312.5 µg/mL	Colistin-resistant strains	Cancer patients.	AB	[113]
Cinnamon bark oil (*Cinnamomum zeylanicum*) and other EOs/Lauraceae	Cinnamaldehyde and Eugenol	EOs MIC: 0.0562–0.225%|(*v*/*v*)	*P. aeruginosa* (PAO1) and MDR *P. aeruginosa* (MDR-PA)	Clinical isolates	AB	[114]
*Cinnnamon cassia*	Cinnamaldehyde (87.6%)	EOs MIC: 281.25 μg/mL	Carbapenemase-producing *K*. *pneumoniae* (KP-KPC) and *S*. *marcescens* (SM-KPC)	Rectal swab and urine sample	AB	[115]
*Cinnnamon cassia*	Cinnamaldehyde (87.6%)	EOs MIC: 17.57 μg/mL	Carbapenem- and polymyxin-resistant *Klebsiella aerogenes*	Nasal swab	AB	[116]
Clove (*Syzygium aromaticum*)/Myrtaceae	Eugenol (96.35%)	EOs MIC: 23.0 to 51.0 μg/mL	MDR *Helicobacter pylori*	Clinical samples	AB, ABF	[117]
	Eugenol (67.4%)	EOs MIC: 200 μg/mL	*Campylobacter jejuni*	Clinical samples	AB, AV	[118]
Clove (*Syzygium aromaticum*)/Myrtaceae, Thyme (*Thymus vulgaris*) chitosan coated emulsions for nose and brain delivery/Lamiaceae	Clove EOs: eugenol (80.1%); Thyme EOs: thymol (44.4%)	Not specified	*S. aureus* subsp. aureus-MSSA, *E. coli*, MRSA, carbapenem-resistant K. pneumoniae (CR-Kp ), carbapenem-resistant *A. baumanni* (CR-Ab), and carbapenem-resistant *P. aeruginosa* (CR-P)	MRSA isolated from skin; CR-Kp isolated from urine; R-Ab clinical strain isolated from sputum; CR-Pa clinical strain isolated from bronchoalveolar lavage	AB	[119]
*Nigella sativa* seed oil/Ranunculaceae	Not specified	Not specified	Methicillin-resistant *S. aureus* (MRSA)	Diabetic patients’ wounds	AB	[120]
*Nigella sativa* seed oil/ Ranunculaceae	Thymoquinone (30–52.6%) and p-cymene (7–25.8%)	EOs MIC: <0.25 μg/mL to 1.0 μg/mL	Methicillin-resistant *S. aureus* (MRSA), and methicillin-resistant CoNS (MRCoNS)	Clinical samples	AB	[121]
(*Coriandrum sativum*)/*Apiaceae*, *Cinnamomum cassia*/*Lauraceae*, *Ziziphora hispanica*/*Lamiaceae*			MDR uropathogenic *E. coli*	Patients with UTIs	AB	[122]
	Not specified	*Cinnamomum cassia* EOs MIC: <5 mg/mL.	*E. coli*, *P. aeruginosa*, *K. pneumoniae* and *P. mirabilis* susceptible and resistant phenotypes	Clinical samples from patients with UTIs	AB	[123]
Eucalyptus (*Eucalyptus camaldulensis*) leaf/Myrtaceae	Patulenol, cryptone, p-cimene, 1,8-cineole, terpinen-4-ol and β-pinene	EOs MIC: 0.5 to 2 μL/mL	MDR *Acinetobacter baumannii*	Wound isolates	AB	[20]
Eucalyptus (*Eucalyptus globulus*), Tea Tree (*Melaleuca alternifolia*), Clove (*Syzygium aromaticum*)/Myrtaceae, Cinnamomum (*Cinnamomum zeylanicum*)/Lauraceae	Not specified	Not specified	*P. aeruginosa* and *S. aureus*	Clinical isolates	ABF	[59]
Geranium (*Pelargonium graveolens* *Ait*)/Geraniaceae	Citronellol (26.7%) and geraniol (13.4%)	EOs MIC vary between 3.0 μL/mL to 10.5 μL/mL	*E. coli*, *C. freundii*, *E. sakazakii*, *E. cloacae*, *P. mirabilis* and *P. aeruginosa* resistant strains	Patients with difficult to heal wounds	AB	[124]
Geranium (*Pelargonium graveolens*)/*Geraniaceae*, *Rosemary* (*Rosemary officinalis*)/*Lamiaceae*, and *Peppermint* (*Mentha piperita*)/*Lamiaceae*		Mint EOs MIC: 2.5–5 μL/mL; Geranium EOs MIC: 5–20 μL/mL;Rosemary EOs MIC: 5–20 μL/mL	XDR colistin-resistant and colistin susceptible *A. baumannii*	Clinical isolates	AB, ABF	[40]
Ginger (*Zingiber officinale*)/Zingiberaceae	9,12-Octadecadienoic acid methyl ester 466 (50.49%) and Hexadecanoic acid methyl ester (38.05%)	EOs MIC: 1.5 mg/mL	*P. aeruginosa* producing extended spectrum β-lactamase (ESβL) enzyme	Wound samples of burn patients	AB	[125]
	Not specified	Not specified	MDR and XDR *E.coli* blaTEM genotypes	Clinical samples from patients with UTIs	AB	[99]
Lemongrass (*Cymbopogon citratus*)	*b-myrcene (57.52%)*	EO MIC ranges between 0.1–3.2% (*v*/*v*)	*Klebsiella pneumoniae*, *Pseudomonas aeruginosa* and *Staphylococcus epidermidis*	Patients with Chronic rhinosinusitis	AB, ABF	[126]
Lemongrass (*Cymbopogon citratus*) topical application/Poacea and other EOs	Citral (60.6%)	EOs MIC: 0.1 mg/mL	*Bacillus thuringiensis, Kytococcus sedentarius, Dermatophilus congolensis*	Pitted keratolysis lesions	AB	[127]
Lemongrass (*Cymbopogon citratus*)/Poaceae, Lavender (*Lavandula angustifolia*)/Lamiaceae, Marjoram (*Origanum majorana*)/Lamiaceae, Peppermint (*Mentha* × *piperita*)/Lamiaceae, Tea Tree (*Melaleuca alternifolia*)/Myrtaceae, and Rosewood (*Rosmarinus officinalis*)/Lamiaceae	Monoterpenes including monoterpene alcohols such as terpinen-4-ol or geraniol; bicyclic monoterpenes such as camphene, borneol, pinenes, sabinene or camphor; acyclic monoterpenoids (or derivatives) such as myrcene, geranyl acetate, citronellol or linalool	Terpinen-4-ol MIC: 0.125–0.5% *v*/*v*; Geraniol MIC: 0.125–1% *v*/*v*	MDR *Burkholderia cepacia* complex	Patients with cystic fibrosis	AB, AE	[41]
Peppermint (*Mentha × piperita*)/Lamiaceae	Monoterpene hydrocarbons(34.23%), monoterpene oxygenates (60.83%)	EOs MIC (*S. aureus*, *E. coli*, and *P. mirabilis*) < 20 mg/mL; EOs MIC *(K. pneumoniae*, *P. aeruginosa*, and *A. baumannii*) > 40 mg/mL	MDR *A. baumannii*, *E. coli*, *K. pneumoniae*, *P. mirabilis*, *P. aeruginosa* and *S. aureus.*	*A. baumannii*: bronchoalveolar lavage samples; *E. coli*: urine and wound secretion; K. pneumoniae: urine and bronchoalveolar lavage; P. mirabilis: wound secretion; *P. aeruginosa:* wound secretion and otic discharge; *S. aureus*: nasal swabs and wound secretion.	AB	[128]
Menthol and Mint (*Mentha longifolia)*		With Ciprofloxacin: 4-fold MIC reduction; With Imipenem: 8-fold MIC reduction; Menthol + Imipenem: up to 16-fold MIC reduction (90% isolates)	Carbapenem-resistant and fluoroquinolones-resistant *A. baumannii*	Clinical isolates	AB, AE	[42]
Oregano EOs (*Origanum vulgare*)/Lamiaceae	Carvacrol (71%)	EOs MIC (*K. pneumoniae* and *S. marcescens*): 0.059% (*v*/*v*)*; EOs MIC* (*A. baumannii*): 0.015% (*v*/*v*).	Carbapenem-resistant *K. pneumoniae and S. marcescens, and A. baumannii*	Rectal swab, urine sample, and nasal swab, respectively	AB	[129]
	Carvacrol (71%)	EOs MIC: 1.75 to 3.50 mg/mL	MDR *A. baumannii*	Clinical isolates	AB	[130]
Oregano and thyme red/Lamiaceae	Oregano Eos: Carvacrol (77.8%); Thyme red oil: thymol (53.3%)	MIC of oregano oil, thyme red oil, carvacrol, and thymol against UPEC were 0.1%, 0.1%, 0.05% *v*/*v* respectively	Uropathogenic *E. coli* O6:H1 strain (UPEC)	Clinical isolates	AB, ABF	[131]
Oregano (*Origanum onites*)/Lamiaceae	Carvacrol (51.4%), linalool (11.2%), p-cymene (8.9%) and γ-terpinene (6.7%)	MIC:1.56–25 µL/mL	Extended spectrum beta lactamase (ESBL) producer, carbapenem resistant *E. coli*	Clinical samples	AB	[132]
Oregano EOs/Lamiaceae	Carvacrol (72.25%)	MIC: 0.08 to 0.64 mg/mL	*A. baumannii*, *P. aeruginosa*, and *MRSA*	Clinical samples from combat casualties	AB, ABF	[35]
Wild oregano/Lamiaceae, Garlic/Amaryllidaceae, Black pepper/Piperaceae	Not specified	Oregano EOs MIC: 0.02–1.25 mg/mL; Garlic EOs MIC: 0.02–40 mg/mL; Black pepper EOs MIC: 0.04–40 mg/mL	*Clostridioides difficile*	Stool specimens of hospitalized patients with diarrhea and CDI	AB, ABF	[133]
Oregano (*Origanum vulgare*)/Lamiaceae, Thyme (*Thymus vulgaris*)/Lamiaceae, Lavender (*Lavandula angustifolia*)/Lamiaceae, Peppermint (*Mentha × piperita*)/Lamiaceae, Tea Tree (*Melaleuca alternifolia*)/Myrtaceae	Carvacrol	Thyme and oregano EOs MIC:256 to 512 μg/mL; Carvacrol EOs MIC: 64 to 256 μg/mL	*Erythromycin-resistant Streptococcus pyogenes [Group A streptococci (GAS)]*	Children with pharyngotonsillitis	AB, AE	[43]
Rosemary (*Rosmarinus officinalis*)/Lamiaceae	1,8-cineole (17.16%), α-pinene (16.95%) and verbenone (15.78%)	MIC: 0.06 to 0.16 ± 0.07 mg/mL	*S. aureus*, *K. pneumoniae*, and *Proteus vulgaris*	Urine samples from patients suspected of UTI	AB	[134]
Rosemary (*Rosmarinus officinalis*)/Lamiaceae, Oregano (*Origanum majorana)/Lamiaceae*, Thyme (*Thymus zygis*)/Lamiaceae, Juniper (*Juniperus communis*) Cupressacea, Ginger (*Zingiber officinale*)/Zingiberaceae	*J. communis* EOs: α-Pinene (47.1%), β-Myrcene (11.7%); Z. officinale: α-Zingiberene (33.1%), β-Sesquiphellandrene (13.5%); *O. majorana* EOs: terpinen-4-ol (25.9%), γ-Terpinene (16.9%), Linalool (10.9%),T. zygis: Linalool (39.7%), Terpinen-4-ol (11.7%); *R. officinalis EOs*: 1,8-Cineole (47.7%), α-Pinene (11.7%),	Rosemary EOs MIC: 1.56–3.125 mg/mL; Thyme EOs MIC: 0.19–0.78 mg/mL; Marjoram EOs MIC: 0.19–0.78 mg/mL.	*E. coli*	Urine samples from patients with clinical symptoms of UTI	AB, ABF	[135]
Tea Tree (*Melaleuca alternifolia*)/Myrtaceae	Terpinen-4-ol (40.4%), γ-terpinene (19.5%), and α-terpinene (7.7%).	EOs MIC (*S. aureus*): 0.048–3.125 mg/mL for the whole essential oil, terpinen-4-ol MIC (*S. aureus*): 0.048–1.52 mg/mL	MRSA	Superficial and deep pus, blood culture and strains of various other specimens (tracheal aspiration, wound)	AB, ABF, AQS	[60]
	-	-	MDR *P. aeruginosa*	Patients with cystic fibrosis	AB, ABF	[136]
	-	-	Carbapenem-resistant *S. marcescens*	Clinical isolates	AB, ABF	[137]
*Thymbra capitata*/Lamiaceae, *Thymus pallescens*/Lamiaceae, White Wormwood (*Artemesia herba-alba*)/Asteraceae	*Thymbra capitata* EO: Carvacrol (58.68%); *Thymus pallescens* EO: Carvacrol (70.22%); *Artemisia herba-alba*: Camphor (34.62%), Chrysanthenone (25.11%)	*T. pallescens* EOs MIC: 0.16 to 0.63 mg/mL	*K. pneumoniae, E. coli,* and *S. aureus.*	Clinical isolates	AB, ABF	[138]
*Thymus daenensis* L., *Origanum vulgare* L./Lamiaceae	Thyme EOs: Carvacrol (40.69%), γ-terpinene (30.28%); Oregano EOs: pulegone (44.31%), 1,8-cineole (17.47%),	Thyme EOs MIC: 0·625–2·5 μL/mL; Oregano EOs MIC: 1·25–5 μL/mL	Fluoroquinolone-resistant *Streptococcus pneumoniae*	Clinical isolates	AB, AB, AE	[96]
Thyme (*Thymus daenensis*), Summer savory (*Satureja hortensis*), Oregano (*Origanum vulgare*)/Lamiaceae	*T. daenensis* EO: carvacol (40.69%) γ-terpinene (30.28%), and α-terpinene (5.52%); S. hortensis EO: thymol (41.28%), γ-terpinene (37.63%), pcymene (12.2%) and α-terpinene (3.52%).	Thyme EOs MIC: 0.625–1.25 μL/mL; *Satureja hortensis* EOs MIC:2.5 μL/mL; Oregano EOs MIC: 2.5–10 μL/mL	*S. pneumoniae*	Clinical isolates	AB, ABF, AQS	[139]
*Thyme* (*Thymus vulgaris*)*/Lamiaceae, Cinnamon (Cinnamomum verum)/Lauraceae, Oregano (Origanum majorana)/Lamiaceae*, and *Clove (Eugenia caryophyllata)/Myrtaceae*	Not specified	Not specified	MDR bacteria	Clinical isolates	AB, ABF, AQS	[44]
*Thyme (Thymus vulgaris)/Lamiaceae, Clove (Eugenia caryophyllata)/Myrtaceae, Oregano (Origanum vulgare)/Lamiaceae,* and other EOs	*Origanum vulgare* EO: carvacrol (71.8%), p-cymene (11.6%); *Thymus vulgaris* EO: Thymol (43.1%), p-cymene (47.9%); Eugenia caryophyllata EO: Eugenol (85%), β-caryophyllene (9%)	Not specified	*B. cepacia* complex	Patients with cystic fibrosis	AB	[140]
Laurel (*Nectandra megapotamica)*/Lauraceae	Caryophyllene oxide (22.3%)	EOs MIC: 36,000 µg/mL	MDR OXA-23-producing *A. baumannii*	Human nasal swab	AB, AV	[141]
Sage (*Salvia fruticosa*, *Salvia officinalis* and *Salvia sclarea*)/Lamiaceae	Not specified	Not specified	Tetracycline resistant *S. epidermidis*	Clinical isolates	AB, AE	[142]
Basil (*Ocimum basilicum)*, Sage (*Salvia officinalis*)/Lamiaceae	Basil EOs: Linalool and (E)-anethole; Sage EOs: α-thujone and camphor		*P. aeruginosa* resistant strains	Urine sample, skin, throat, eye, ear, and wound swabs	AB, ABF	[143]
*Pituranthos chloranthus*, *Teucruim ramosissimum, Mastic (Pistacia lentiscus*) areal parts/Apiaceae	sabinene (29.6%), limonene (16.65%), terpinen-4-ol (15.55%)	*Pistacia chloranthus* EOs MIC: 0.25–0.5 mg/mL; *Teucrium ramosissimum* EOs MIC: 0.25–1 mg/mL; *Pistacia lentiscus* EOs MIC: 0.125–1 mg/mL against MRSA. MIC: 1 mg/mL against *E. coli* and *Acinetobacter baumannii*	*E. coli* (ESBL), ceftazidime-resistant *A. baumannii,* and MRSA	Patients with UTI	AB	[12]
Java plum *(Syzygium cumini)* leaves/Myrtaceae	α-pinene (53.21%)	EOs MIC (*E. coli*): 512 µg/mL	*E. coli, P. aeruginosa* and *S. aureus*, and clinical isolates MDR *E. coli, P. aeruginosa* and *S. aureus*	Laboratory and clinical isolates	AB	[144]
*Mentha pulegium*/Lamiaceae, White Wormwood (*Artemisia herba alba*)/Asteraceae	*M. pulegium* EO: pulegone (74.8%) and neoisomenthol (10.0%); *A. herba* albaEO: camphor (32.0%), α-thujone (13.7%), 1,8-cineole (9.8%), β-thujone (5.0%), bornéol (3.8%), camphene (3.6%), and p-cymene (2.1%).	*M. pulegium* EOs MIC: 1.2 to 9.4 µL/mL; *A. herba alb* EOs MIC: 1.2 to 4.7 µL/mL.	*Listeria innocua, S. aureus* and *MRSA; E. coli, P.aeruginosa* and *Imipenem-resistant A. baumannii*, producing OXA-23 enzyme and resistant to cefotaxime (CTX) and cefepime (FEP).	*Listeria innocua, S. aureus* and MRSA were isolated from pus; *E. coli*, *P.aeruginosa*, and Imipenem-resistant *A. baumannii* isolated from the catheter.	AB	[145]
*Melaleuca alternifolia*/*Theaceae*, *Eucalyptus globulus*/*Theaceae*; *Mentha* × *piperita*/*Lamiacea*, and *Thymus vulgaris/Lamiacea*		Tea Tree EOs MIC: 0.5–4 µg/mL for *K. pneumoniae* (55%), *P. aeruginosa* (45%), and *E. coli* (95%)	*ESBL E. coli* and *K. pneumoniae*, metallo-beta-lactamase (MBL)-producing *P. aeruginosa* and carbapenemase (KPC)-producing *K. pneumoniae.*	Urine, rectal swabs, and respiratory tracts	AB, ABF	[146]
		Thyme EOs MIC: 1–16 µg/mL for *K. pneumoniae* (90%), *P. aeruginosa* (90%), and *E. coli* (85%)				
		Peppermint EOs MIC: 8–128 µg/mL for *K. pneumoniae* (90%), *P. aeruginosa* (80%), and *E. coli* (95%)				
		Eucalyptus EOs MIC: 32–64 µg/mL for *K. pneumoniae* (90%)*, P. aeruginosa* (80%), and *E. coli* (95%)				

**Table 2:** Methods for assessing the antibacterial mechanisms and activity of plant EOs.

Category	Description	Subcategory	References *
Basic Antimicrobial Activity Methods			
Agar Disk Diffusion	Screens for antimicrobial activity by measuring inhibition zones around disks impregnated with EOs, assessing the inhibition of bacterial growth on agar plates.	Disk Diffusion Method	[39,42,52,111,114,142,143,147,155,156,157]
Agar Dilution	Determines antimicrobial activity by incorporating EOs into agar and measuring bacterial growth at various concentrations to find the minimum inhibitory concentration (MIC).	MIC Determination Methods	[39,42,52,111,114,142,143,147,155,156]
Broth Micro-dilution	Determines MIC and minimum effective concentration (MEC) in liquid media by evaluating bacterial growth in diluted EOs concentrations.	MIC Determination Methods	[2,42,85,111,115,119,125,137,143,146,148,158,159,160]
Time Kill Assay	Measures bacterial viability at various time points post-exposure to EOs to assess bactericidal or bacteriostatic effects over time.	Time-Kill Method	[20,52,64,89,125,137,159,161,162]
Turbidimetry	Assesses antimicrobial activity by measuring turbidity changes in liquid cultures, indicating bacterial growth or inhibition.	Turbidimetric Method	[163]
Bioautography	Detects antimicrobial activity of EOCs using a chromatographic technique with a biological assay to identify active compounds based on inhibition zones.	Bioautographic Method	[20,114,164]
Broth Dilution Volatilization Assay	Combines broth microdilution and vapor-phase methods to assess antimicrobial activity of EOs’ volatile compounds in both liquid and vapor phases.	Volatilization Assay	[165,166]
Vapor Assay	Evaluates EOs’ antimicrobial activity in their vapor phase by exposing bacteria to vapor and assessing growth inhibition or bactericidal effects.	Volatilization Assay	[161,167]
Microscopy and Imaging Techniques			
Scanning Electron Microscopy (SEM)	Provides high-resolution, three-dimensional images of biofilm structure and bacterial surfaces to assess EOs’ impact on biofilm integrity.	Imaging Technique	[35,137,148,149,150,168,169,170,171]
Transmission Electron Microscopy (TEM)	Delivers high-resolution images of microbial cell ultrastructure to observe internal cellular effects of EOs.	Imaging Technique	[125,172]
Light microscopy	Uses optical lenses and visible light to observe and analyze bacterial morphology and the effects of EOs on biofilm formation and disruption.	Imaging Technique	[173,174]
Confocal Laser Scanning Microscopy (CLSM)	Provides high-resolution, three-dimensional images of biofilm structure and EOs’ effects on biofilm formation and disruption.	Biofilm Inhibition and Disruption Assays	[142,149]
Advanced Analytical Methods			
Flow cytometry	Examines bacterial cell viability and vitality post-exposure to EOs, providing rapid, sensitive single-cell analyses.	Single-cell analysis techniques	[85,143]
Raman spectroscopy	Quantifies EOCs and detects interactions with bacterial cells by measuring vibrational spectra, offering insights into molecular composition.	Single-cell analysis techniques	[175,176]
Liquid Chromatography-Mass Spectrometry (LC-MS/MS)	Identifies and quantifies proteins expressed in response to EOs, revealing molecular mechanisms and potential targets.	Mass Spectrometry-Based Proteomics	[177]
X-Ray Diffraction (XRD)	Provides structural information on antimicrobial agents by analyzing the diffraction patterns of X-rays.	Analytical Methods	[172]
Fourier Transform Infrared Spectroscopy (FTIR)	Analyzes functional groups and molecular interactions by detecting vibrational modes of molecules.	Analytical Methods	[172]
Attenuated Total Reflectance Infrared (ATR-IR) Spectroscopy	Analyzes chemical composition and interactions by detecting changes in functional groups and molecular bonds.	Analytical Methods	[178]
Dynamic Light Scattering (DLS)	Measures the size distribution and stability of nanoparticles in a solution by analyzing fluctuations in scattered light intensity.	Analytical Methods	[161]
Spectrofluorometry	Measures fluorescence emitted by samples to analyze interactions, quantify fluorescent probes, and investigate the behavior of EOs with bacterial cells.	Analytical Methods	[179]
Biofilm Analysis Methods			
XTT Viability Assay	Measures metabolic activity and viability of biofilms exposed to EOs by quantifying reduction of XTT dye.	Biofilm Formation and Viability assays	[180]
Microtiter Plate Method (MtP)	Quantifies biofilm formation, bacterial growth, and metabolic activity post-EO exposure, allowing high-throughput analysis.	Biofilm Formation and Viability assays	[111,137,170,174]
Congo Red Agar Method (CRA)	Visualizes biofilm production using Congo red dye; black colonies indicate biofilm production, while pink colonies suggest weak formation.	Biofilm Formation and Viability assays	[59]
Antibiofilm Activity Assay	Assesses the effectiveness of EOs in disrupting established biofilms using the Alamar Blue assay for bacterial viability.	Biofilm Formation and Viability assays	[127,142,174,181]
Surface Coating with Biofilm Inhibitors	Evaluates the efficacy of EOs in preventing microbial adhesion and biofilm formation through cell membrane surface treatments.	Biofilm Formation and Viability assays	[182]
Alginate Assay	Measures alginate production, a key extracellular polymeric substance, to assess EOs’ impact on biofilm matrix integrity.	Biofilm Formation and Viability assays	[73]
Crystal Violet Staining for Biofilm Quantification	Quantifies biofilm biomass by staining adhered cells with crystal violet, measuring reduction in biofilm biomass due to EOs.	Biofilm Formation and Viability assays	[12,69,71,73,91,137]
Extracellular polymeric substances (EPS) Inhibition Assays	Evaluates EOs’ effect on EPS to assess their ability to disrupt biofilm formation and stability.	Biofilm Inhibition and Disruption Assays	[58]
Ethidium Bromide Cartwheel (EtBr-CW) Method	Visualizes and quantifies biofilm formation by staining with ethidium bromide, allowing assessment of EOs’ effects on biofilm.	Biofilm Formation and Viability assays	[54,74,158]
DNA Fixation with Ethanol	Preserves DNA integrity for analyzing changes due to EOs, assessing their impact on biofilm structure and stability.	Biofilm Formation and Viability assays	[49]
Cell Viability and Damage Assessment			
Live/Dead Assay	Utilizes fluorescent dyes to measure bacterial viability, distinguishing live cells from dead ones to evaluate EOs’ impact.	Biofilm Formation and Viability assays	[155]
MTT Viability Assay	Evaluates cytotoxicity to ensure the safety of EOs for therapeutic applications by measuring metabolic activity.	Cytotoxicity Evaluation	[137,150,171]
Resazurin Microplate Assay	Assesses cell viability and metabolic activity by measuring the reduction of resazurin to resofurin.	Analytical Methods	[52,142,183]
Molecular and Genetic Analysis			
Real-Time Quantitative PCR (RT-qPCR)	Quantifies mRNA levels to study gene regulation in response to EOs, revealing molecular responses and mechanisms.	Molecular Techniques	[96,119,127,137,170]
Proteomic Expression Validation through qRT-PCR	Confirms changes in protein expression due to EOs, validating proteomic data and revealing antimicrobial mechanisms.	Molecular Techniques	[52,89]
Detection of adeABC genes	Identifies efflux pump genes associated with antibiotic resistance to assess the impact of EOs on resistance mechanisms.	Molecular Techniques	[42]
Multiplex PCR	Allows simultaneous amplification of multiple target DNA sequences, detecting various bacterial genes or virulence factors in response to EOs.	Molecular Techniques	[184]
RNA isolation	Extracts and purifies RNA for subsequent analyses, such as RT-qPCR, to investigate gene expression changes following EOs exposure.	Molecular Techniques	[111,149]
Membrane and Cell Surface Analysis			
Outer Membrane Permeability Assay	Evaluates the disruption of the outer membrane of Gram-negative bacteria by EOs, facilitating increased permeability and antibacterial effects.	Analytical Methods	[52,141]
zeta potential measurement	Assesses changes in bacterial surface charge upon exposure to EOs, quantifying effects on bacterial cell surface properties.	Analytical Methods	[52,136,185]
Ethidium Bromide Influx/Efflux Assay	Measures the influx and efflux of ethidium bromide in bacteria to assess the impact of EOs on bacterial efflux pump activity and membrane permeability.	Analytical Methods	[96,119,127]
ATP Concentration Determination	Measures intracellular ATP levels to assess cell viability and metabolic activity following exposure to EOs.	Analytical Methods	[92]
Membrane Integrity Assay	Evaluates the integrity of bacterial cell membranes by detecting leakage of intracellular components or uptake of membrane-impermeable dyes.	Analytical Methods	[64,186]
Bacterial Virulence Assessment			
Bioluminescence Expression Anti-QS Assay	Uses bioluminescent reporter strains to evaluate the effect of EOs on QS pathways and bacterial communication.	Quorum Sensing (QS) Inhibition Bioassay:	[10,141]
Violacein Inhibition Assay	Assesses interference with QS using *C. violaceum* strain CV026 and changes in violacein production.	Quorum Sensing (QS) Inhibition Bioassay:	[149,187,188]
Skim Milk Agar Assay	Assesses protease activity by observing clear zones around colonies on agar plates with skim milk, indicating EOs’ ability to inhibit protease production.	Assays of Virulence Factors	[189]
Azocasein assay	Measures protease activity through degradation of azocasein, providing insights into EOs’ impact on protease activity and bacterial virulence.	Assays of Virulence Factors	[142]
Swarming Motility	Evaluates bacterial migration across solid surfaces to assess EOs’ impact on motility and pathogenicity.	Assays of Virulence Factors	[142,149]
Hemagglutination Assay	Assesses bacterial migration across surfaces to evaluate EOs’ effects on motility and pathogenicity.	Assays of Virulence Factors	[146]
Advanced Systems and Models			
Microfluidic Systems	Facilitates high-throughput screening, real-time monitoring, and precise control to study EO nanoemulsions against pathogenic bacteria.	Microfluidics and Lab-on-a-Chip (LOC) Devices.	[190,191]
Computational Fluid Dynamics (CFD) Models	Models fluid flow and interactions to investigate kinetics of antibacterial activity and parameters affecting bacterial lysis.	Microfluidics and Lab-on-a-Chip (LOC) Devices.	[16,89,191]
Organs-on-Chips	Simulates human organ functions using microfluidic devices to study EOs’ effects on human microbiomes or pathogen-host interactions.	Advanced Cell-Based Assays	[55,173,190,191]
Synergy Studies			
Fractional Inhibitory Concentration Index (FICI) Calculation	Quantifies the degree of synergy or antagonism between EOs and antibiotics by calculating the FICI.	Synergistic effect determination	[40,114,115,136,138,147,151,192]
Checkerboard Assay	Evaluates the synergistic effects of combinations of EOs and antibiotics by assessing their combined antimicrobial activity.	Synergistic effect determination	[20,43,52,85,89,96,114,115,142,147,158,193,194,195]

* The listed references represent the studies included in this review.

## Data Availability

The original contributions presented in this study are included in the article/Supplementary Material. Further inquiries can be directed to the corresponding author.

## Supplementary Materials

The following supporting information can be downloaded at: https://www.mdpi.com/article/10.3390/antibiotics14121250/s1, Table S1. Antibacterial efficacy of selected plant essential oils against resistant clinical pathogens.

## Abbreviations

The following abbreviations are used in this manuscript:

ABC	ATP-binding cassette
AFM	Atomic force microscopy
AIPs	Autoinducing peptides
AIs	Autoinducers
AMR	Antimicrobial resistance
AST	Antimicrobial susceptibility testing
BMD	Broth microdilution
BPPL	Bacterial Priority Pathogens List
CAC	Codex Alimentarius Commission
CFD	Computational fluid dynamics
CHT	Chitosan-based systems
CLSI	Clinical and Laboratory Standards Institute
CLSM	Confocal laser scanning microscopy
CRKP	carbapenem-resistant *K. pneumoniae*
DESI	Desorption electrospray ionization
EMA	European Medicines Agency
EOCs	Plant EOs and their components
EOs	Plant essential oils
ESBLs	Extended-spectrum β-lactamases
EtBr-CW	Ethidium bromide cartwheel
EUCAST	and the European Committee on Antimicrobial Susceptibility Testing
FAO	Food and Agriculture Organization
FBECI	Fractional biofilm eradication concentration index
FDA	Food and Drug Administration
FICI	Fractional inhibitory concentration index
FID	Flame ionization detector
FTIR	Fourier transform infrared spectroscopy
GACP	Good Agricultural and Collection Practices
GC	gas chromatography
GNB	Gram-negative bacteria
GPB	Gram-positive bacteria
ISO	International Organization for Standardization
LOC	Lab-on-a-chip
LPS	lipopolysaccharides
MATE	Multidrug and toxic compound extrusion
MBC	minimum bactericidal concentrations
MBL	Metallo-beta-lactamase
MDR	multidrug-resistant
MeSH	Medical Subject Headings
MF	Major facilitator
MIC	Minimum inhibitory concentrations
MOCS	Substance with more than one constituent
MRCoNS	Methicillin-resistant coagulase-negative staphylococci
MRSA	Methicillin resistant *Staphylococcus aureus*
MS	Mass spectrometer
NDS	Nanostructured delivery systems
NMR	Nuclear magnetic resonance
OmpF	Outer membrane protein F
PCR	Polymerase chain reaction
PEB	protein energy binding
QS	Quorum sensing
RND	Resistance nodulation division
ROS	Reactive oxygen species
SEM	Scanning electron microscopy
SMR	Staphylococcal multi-resistance
TEM	Transmission electron microscopy
UTI	Urinary tract infection
XDR	extensively drug-resistant
XRD	X-ray diffraction

## Appendix A

### Appendix A.1. Current Plant EO Extraction Methods

**Methods**	**Process**	**Advantages**	**Disadvantages**
Conventional methods			
Cold-press extraction	-Used extensively for extraction of citrus peel EOs -Predominantly mechanical process which compresses peels or whole fruits to release the EOs -Released oils are washed from the resultant paste using water -Water may be evaporated to produce concentrated EOs	-Minimal heat exposure -Preserves natural oil properties -Suitable for citrus fruits	▪ Contains non-volatile impurities, coumarins, and pigments ▪ Limited application (only suitable for citrus fruit peels) ▪ Low purity and yield
Hydrodistillation (HD)	-Plant material is placed into water and brought to boiling (100 °C) -Evaporated components are captured by condensation -Components are separated from residual water	-Extracts compounds with boiling points below 100 °C -Faster process than steam distillation -Convenient set-up and operation -Low cost -Efficient extraction due to better penetration	-Limited extraction of high boiling point compounds -Lower yield than steam distillation -Susceptibility to -hydrolysis reactions -High energy consumption -Prolonged process time -Potential volatile losses -Thermal degradation of sensitive compounds -High carbon dioxide emissions
Steam distillation (SD)	-Plant material is exposed to steam at 250–350 °C -EOs components evaporate and are captured in a condenser -Components separated from residual water	-Widely used at industrial scale -Lower susceptibility to hydrolysis than HD -Higher yields than HD -Convenient process control	-Thermal degradation and structural alterations, especially for monoterpenes, due to high temperature
Solvent extraction	-Plant material is mixed with a solvent (ethanol, methanol, acetone, ether, or hexane) -Mixture is heated to less than 100 °C -Extract is filtered to remove plant material -EOs- is concentrated by evaporation of solvent, often under vacuum	-Simple method for EOs extraction	-Potential solvent contamination and impurities -Volatile losses during solvent evaporation -Environmental hazards from solvent waste -Extraction yield and quality depend on numerous factors (solvent type, temperature, extraction cycles, vessel design, raw materials particle size)
Advanced Methods			
Omic-assisted hydrodistillation (OAHD)-modern route	-Electrical current passed through mixture of plant material and water -Plant material acts as resistor, converting electrical energy into heat via Joule effect -Internal heating causes release of essential oils -Oils collected through process similar to traditional HD	-Overcomes HD limitations -Rapid extraction -Minimizes volatile losses -Energy-efficient -Improved process control -Cost-effective	-Electrical conductivity concerns -Operational safety challenges -High capital investment required
Microwave-assisted hydrodistillation (MAHD)	-Advanced HD technique utilizing a microwave oven -Based on dielectric heating from microwaves for effective and selective heating -Modified microwave oven connected to Clevenger apparatus for lab-scale distillation -Microwave energy converted to heat energy in water due to high dielectric properties -Heat transferred to plant materials	-Rapid process	-Transition to coaxial MAHD recommended for better cost, scalability, safety, and cost-effectiveness
Microwave steam distillation (MSD)	-Microwave oven connected to reactor containing plant materials or standard steam distillation apparatus -Saturated steam generated and passed through plant material in microwave zone -Combination of steam and direct microwave heating causes rapid release of essential oils -Oils collected through condensation	-Effective heating -Selective extraction -High extraction efficiency -Reduced energy consumption -Reduced extraction time -Less structural alteration of chemical compounds due to lower overall heat exposure	
Turbo Hydrodistillation	-Mixture of water and plant materials constantly stirred at specific rpm while undergoing hydrodistillation -Agitation enhances extraction process by increasing contact between plant material and water	-Improved extraction efficiency	-Potential degradation of sensitive compounds due to intense agitation/stirring
Salt-Assisted Hydrodistillation	-Plant materials mixed with water and NaCl (salt) before conventional hydrodistillation -Salt alters polarity of water, potentially improving extraction efficiency	-Faster processing	-Increased processing cost and complexity
Enzyme-Assisted Hydrodistillation	-Plant materials mixed with water and specific enzyme -Mixture incubated at particular temperature with stirring before hydrodistillation -Enzymes break down cell walls, potentially releasing more essential oils	-Higher yields	-Salt residue removal required
Micelle-Mediated Hydrodistillation	-Plant materials mixed with aqueous surfactant solution (e.g., 10% Tween 40) before hydrodistillation -Surfactant forms micelles that can encapsulate essential oil components	-Milder extraction conditions than HD -Reduced or eliminated need for added water/solvents in some techniques	-High enzyme costs -Careful selection and optimization needed for different plant materials -Added chemical complexity and cost from surfactant use -Environmental concerns with surfactant disposal.
Solvent-Free Microwave Assisted Extraction (SFMAE)	-Plant materials placed directly in microwave extraction vessel without added solvents or water -Microwaves rapidly heat internal plant water, causing cells to expand and rupture -Released oils are vaporized, then condensed and collected	-Increased extraction kinetics compared to MAHD -Elimination of added solvents or water -Reduced risk of hydrolysis of essential oil components -Potentially higher quality of extracted oils due to minimal water interaction.	-Requires specialized microwave equipment, increasing capital costs -Potential for uneven heating and hot spots in plant material -Potential for uneven heating and hot spots in plant material
Microwave Hydrodiffusion and Gravity (MHG)	-Plant material subjected to microwave energy, heating internal water molecules and causing thermal stress -Leads to rupture of oil glands -EOs drain due to gravity (not evaporated) -EOs are collected at bottom of apparatus	-Improved efficiency, potentially higher quality oils -Reduced processing time and minimal water use.	-Requires specialized microwave equipment -Complex gravity drainage setup compared to traditional condensation -Risk of extract contamination if drainage not properly controlled
Microwave-assisted extraction (MAE)	-Can be performed with or without solvents -Water or solvent added to plant material exposed to microwaves -Heated liquid penetrates plant material and extracts EOs -Liquid/EO mixture evaporated to produce concentrated EOs -Yields affected by microwave power, time, and solvent quality/quantity	-Rapid extraction -Reduced solvent consumption -High yields -Suitable for thermally sensitive compounds -Disruption of weak hydrogen bounds -Environmentally friendly -Various techniques available.	-Limited to small-scale applications -High energy consumption
Ultrasound-assisted extraction (UAE)	-Uses sound waves between 20 kHz and 2000 kHz to cause acoustic cavitation in solvent -Sound waves rupture plant cell walls releasing EOs -Can use range of solvents at temperatures from ambient to 90 °C	-Rapid extraction -Reduced solvent consumption -High yields -Suitable for thermally sensitive compounds -Disruption of weak hydrogen bounds; Environmentally friendly; Various techniques available.	-Limited to small-scale applications -High equipment cost.
Supercritical Fluid Extraction	-Uses properties of both liquid and gaseous phase at critical point -Supercritical fluid passed through plant material repeatedly to extract EOs -Extracted EOs removed from supercritical fluid by decompression -Gas captured for reuse -Efficacy affected by matrix nature, particle size, and water content -Carbon dioxide widely used (critical conditions: 31.1 °C and 7.38 MPa)	-Rapid extraction -Selective extraction -High yields -Environmentally friendly -Low operating cost -High extraction efficiency -Fractionation capability; -Health and safety benefits of using supercritical carbon dioxide -Beneficial chemical properties such as high diffusivity, low viscosity, tunable density and dielectric constant.	-Risk of carbon dioxide retention in the operator’s blood -Challenges in high-pressure industrial operations
Molecular distillation	-Operates under high vacuum and low temperature -Plant extract spread in thin film on heated surface -Molecules with lower boiling points evaporate first and are collected on cooled surface -Allows separation and concentration of specific compounds	-Ability to fractionate and concentrate valuable essential oil components -Potential for high purity extracts/fractions -Mild conditions protect thermally labile compounds	-Added complexity and equipment requirements -Multiple distillation steps required -Relatively low throughput may limit scalability
Fractional distillation	-EOs heated in column -As temperature increases, different compounds vaporize at respective boiling points -Vapors rise through column and are collected at different levels based on volatility -Allows separation of various oil components		

The table is adapted from references [260,261,262].

### Appendix A.2. Comparison Between Conventional and Advanced Methods of EOs Extraction

**Description**	**Conventional Methods**	**Advanced Methods**
Methods	Hydrodistillation, steam distillation, cold pressing, solvent extraction	Microwave-assisted, ultrasound-assisted, enzyme-assisted, ohmic-assisted, membrane-assisted extraction
Industrial Use	Widely used, especially hydrodistillation and steam distillation	Gaining traction due to advantages over conventional methods
Energy Consumption	High	Generally lower
Extraction Efficiency	Lower	Higher
Extraction Rate	Slower	Faster
Effect on Heat-Sensitive Compounds	Can be detrimental	Generally milder, better preservation
Volatile Compound Loss	Potential for significant loss	Minimal loss
Environmental Impact	Higher (more energy, potential toxic residues)	Lower (reduced energy, less or no solvent use, lower CO_2_ emissions)
Solvent Use	Some methods require solvents	Reduced or no solvent use in many techniques
Selectivity	Lower	Higher selectivity for targeted compounds
Complexity	Simpler, well-established	More complex, may require optimization
Cost	Lower initial costs, but potentially higher operating costs	Higher initial costs (equipment), but potentially lower operating costs
Quality and Purity of EOs	Can be affected by heat and processing	Generally higher
Research and Development Needs	Well-established	Require more R&D for optimization
Flexibility	Less flexible, more standardized	
