# Supplementary material for: Essential Oils as Antimicrobial Agents Against WHO Priority Bacterial Pathogens: A Strategic Review of In Vitro Clinical Efficacy, Innovations and Research Gaps
Source: Antibiotics (Basel). 2025 Dec 10;14(12):1250. doi: 10.3390/antibiotics14121250 (PMC12729739; doi:10.3390/antibiotics14121250)
Supplement: Supplementary file 1 [file antibiotics-14-01250-s001.zip › antibiotics-3816977-supplementary.pdf]

Supplementary Table S1. Antibacterial efficacy of selected plant essential oils- against resistant clinical pathogens

Essential Oils Proprietary Name (Species)/ Family	Composition	MIC/MBC	Synergism (FICI ≤0.5)	Additive (FICI ≥0.5)	Bacteria	Isolated from	Antimicrobial mechanism					Important findings	Ref.
							Antibacterial	Anti-biofilm	Anti-efflux	Anti-virulence	Anti-QS		
Cinnamon and cinnamaldehyde / Lauraceae		EOs MIC: 32 µl/ml ; Cinnamaldehyde MIC: 0.00002 to 0.03 µl/ml			*E. coli* carrying *pks* gene	Colon cancer patients, inflammatory bowel disease and healthy subjects	X	X				Downregulates the clbB gene expression; reduces mcr-1 gene expression in colistin-resistant *P. mirabilis* and *E. coli* isolates.	[111]
	Cinnamaldehyde (78.1%), benzyl alcohol (16.67%), linalyl iso-valerate (2.6%), eugenol (1.5%), and β-caryophyllene (1.13%).	EOs MIC: 0.0562–0.225 µl/ml; EOs MBC: 0.1125–0.225 µl/ml			Pan and Extensive Drug-Resistant *P. aeruginosa*	Burn wounds and urine samples	X		X				[112]
Cinnamon, Thyme, Eucalyptus / Lauraceae		EOs MIC (Resistant strains): 4.88 to 312.5 µg/ml			Colistin-resistant strains	Cancer patients.	X					Cinnamon oil showed the highest activity against colistin-resistant strains; reduced mcr-1 gene expression by 20–35-fold in colistin-resistant *P. mirabilis* and *E. coli.*	[113]
Ceylon cinnamon (*Cinnamomum verum*) / Lauraceae				Meropenem	*K.pneumoniae* BAA-1705		X						[147]
		EOs MIC (*P.aeruginosa*): 1 µl/ml			*P.aeruginosa* PAO1 (GFP-PAO1) and *E. coli* pJN105L pSC11		X	X			X	Inhibited QS and biofilm formation at low concentrations (0.1–0.2 µL/mL) by reducing extracellular DNA and EPS production.	[148]

	Trans-cinnamaldehyde (72.81%), benzyl alcohol (12.5%), eugenol (6.57%), coumarin (1.76%), α-terpineol (1.62%), andlLinalool (1.37%).		Piperacillin	Beta-lactamase TEM-1 plasmid-conferred *Escherichia coli* J53 R1		X			X	Reversed *E. coli J53* R1 resistance to piperacillin by modifying the outer membrane permeability and inhibiting bacterial QS.	[152]
	Cinnamaldehyde (64.49%) , eugenol (16.57%), linalool (4.82%), α-pinene (3.02%), and limonene (2.53%).			Enterohemorrhagic *E. coli* O157: H7 (EHEC) and *P. aeruginosa* PAO1		X	X	X		Reduced pyocyanin and PQS production, swarming motility, and hemolytic activity *of P. aeruginosa*.	[149]
		EO MIC (*K. pneumoniae*) : 0.08% (v/v)		*KPC-producing K. pneumoniae*		X				Induced oxidative stress in KPC-KP cells, disrupting bacterial membranes through interaction with the phospholipid bilayer and impairing membrane repair systems.	[185]
	Eugenol (77%), benzyl benzoate (5%), trans caryophyllene (3%), eugenol acetate (3%) and linalool (2%).	EOs MIC: 0.5 mg/ml ; EOs MBC: 1.0 mg/mL		*P. aeruginosa* (ATCC 25619)*, S. aureus* (ATCC 29213) and *K. pneumoniae* (ATCC 13883)		X	X			C. verum leaf EO vapor exhibited antibacterial effects against planktonic *P. aeruginosa, S. aureus, and K. pneumoniae*. Structural changes in preformed biofilms included cell wall damage, cell shrinkage, and intracellular leakage.	[150]
Cinnamon bark oil (*Cinnamomum zeylanicum*) and other EOs / Lauraceae	Cinnamaldehyde (60%-70%), eugenol.	EOs MIC: 0.0562–0.225 % |(v/v) ; EOs MBC: 0.1125–1.8 % (v/v)	Colistin	*P. aeruginosa* (PAO1) and MDR *P. aeruginosa* (MDR-PA)	Clinically isolated multidrug-resistant P. aeruginosa (MDR-PA)	X				Cinnamaldehyde demonstrated better activity than eugenol against *P. aeruginosa.* Cinnamon bark oil and cinnamaldehyde combined with colistin exhibited synergistic activity rates of 16.7% and 10%, respectively, against MDR *P. aeruginosa* strains.	[114]
Cassia (*Cinnamomum cassia*) / Lauraceae		EOs MIC : 19.53 µg/ml	Streptomycin	MDR P. aeruginosa ATCC 27853		X					[151]
		EOs MIC: 4.88 µg/ml	Chloramphenicol	MDR *E. coli* ATCC 25922							

			Ampicillin ; Chloramphenicol	MDR *S. aureus* ATCC 25923						
	Cinnamaldehyde (87.6%), α-humulene (3.1%), γ-elemene (2.5%), borneol (1.5%), cinnamic acid (0.7%), benzaldehyde (0.5%), eugenol (0.4%), and other minor components (3.7%)	EOs MIC:281.25 μg/ml	*Polymyxin B*	Carbapenemase-producing *K. pneumoniae* (KP-KPC) and *S. marcescens* (SM-KPC)	Rectal swab and urine sample	X			The EOs exhibited strong synergistic activity with polymyxin B, reducing the required dosage by 320-fold while maintaining efficacy and minimizing toxic side effects.	[115]
		EOs MIC: 17.57 μg/ml		Carbapenem- and polymyxin-resistant *Klebsiella aerogenes*	Nasal swab	X			The EOs damaged bacterial membranes in a concentration-dependent manner, reduced membrane negative charge, and inhibited arnB gene overexpression. It was effective against carbapenem- and polymyxin-resistant *K. aerogenes* at a low concentration (0.0019% v/v).	[115]
Clove (*Syzygium aromaticum*) / Myrtaceae	Eugenol is the predominant component.	EOs MIC: 23.0 to 51.0 μg/ml		MDR *Helicobacter pylori*	Clinical samples	X	X		Suppressed biofilm formation by 49.32% at 25 μg/ml and 73.21% at 50 μg/ml against H. pylori.	[117]
		EOs MIC: 200 μg/ml; EOs MBC:800 μg/ml		*Campylobacter jejuni* NCTC 11168	Clinical samples	X		X		[118]
Clove, Bay, Pimento berry / Myrtaceae	Eugenol is the predominant component.Myrcene, chavicol, methyleugenol, and β-caryophyllene (>7.8%).			Enterohemorrhagic *E. coli* O157:H7 (EHEC)			X	X	Showed potent antibiofilm activity against EHEC without affecting planktonic growth, reducing the risk of developing drug resistance.	[119]

Clove (Syzygium aromaticum) / Myrtaceae, Thyme (*Thymus vulgaris*) /Lamiaceae	Clove EOs: eugenol (80.1%), eugenol acetate (9.9%) and β-caryophyllene (8.7%). Thyme EOs:thymol (44.4%),o-cymene (18.2%) and linalool (7.5%).		*S. aureus* subsp. aureus-MSSA (ATCC 29213), *E. coli* (ATCC 25922), MRSA , carbapenem-resistant K. pneumoniae (CR-Kp), carbapenem-resistant *A. baumanni (*CR-Ab), and carbapenem-resistant *P. aeruginosa* (CR-P)	MRSA isolated from skin; CR-Kp isolated from urine; R-Ab clinical strain isolated from sputum; CR-Pa clinical strain isolated from bronchoalveolar lavage	X			EOs were formulated in chitosan coated nano-emulsions	[59]
Nigella sativa / Ranunculaceae			*S. aureus*	Diabetic patienst wounds	X				[124]
Black cumin (*Nigella sativa*) / Ranunculaceae	Thymoquinone (52.6%), p-cymene (25.8%),α-thujene (10.5%), β-pinene (3%), α-pinene (2.8%).		*S. aureus, B. cereus,* and *S. typhimurium*		X			Nigella sativa EOs microemulsion exerted superior antibacterial activity compared with ceftriaxone against *S. Typhimurium* and comparable activity against *E. coli*	[40]
Black cumin (Nigella sativa) / Ranunculaceae		EOs MIC: <0.25 µg/ml to 1.0 µg/ml	Methicillin-resistant S. aureus (MRSA), and methicillin-resistant CoNS (MRCoNS)	Clinical samples	X				[126]
	Thymoquinone, carvacrol, and p-cymene.	*Nigella Sativa* EOs MIC (*S. aureus*): 0.03125% ; Thymoquinone MIC (both Ttested strains): 0.0625 mM ; Carvacrol MIC (*S. aureus*): 1 mM; Carvacrol MIC MRSA: 0.5 mM.	*S. aureus* ATCC 25923 and *methicillin and ofloxacin resistant S. aureus* MRSA 272123	Clinical samples	X	X	X	Demonstrated antimicrobial, antibiotic resistance-modulating, and efflux pump-inhibiting activities against S. aureus;<br>Acted as a potent antibacterial agent against nearly all tested strains, with a more pronounced effect on *E. coli (K12).*	[127]

	O-Cymene (37.82%), Carvacrol (17.68%), α-Pinene (10.09%), Trans-sabinene hydrate (9.90%), and 4-Terpineol (7.15%).	EOs MIC (*E. coli K12*): 1.34 µg/ml		Drug-resistant clinical strains including *S. aureus* ATCC 6633), *E. col*i, K12; *B. subtilis (*DSM 6333), *P. mirabilis* (ATCC 29906)	X		Exhibited a more pronouncing effect on *E. coli (K12)* compared with other tested bacteria	[39]
Black cumin (*Nigella sa*tiva) / Ranunculaceae				*S.aureus* (MTCC 9542) and *V. harveyii* (MTCC 152 7771)	X	X	NsEO-AuNPs showed higher antibacterial activity against *S. aureus* (16 mm inhibition zone) than V. harveyii (5 mm inhibition zone) at a concentration of 10 µg/mL.	[41]
Coriander EOs / Apiaceae				MDR uropathogenic *E. coli*	X			[128]
	Linalool (70.11%) is the predominant component.	Coriander EOs MIC: 5.44 µg/ mL; Linalool MIC: 5.36 µg/ ml	Amoxicillin and gentamycin	MRSA strains	X		Increased antibiotic susceptibility and reversed antibiotic resistance in S. aureus and P. aeruginosa.	[42]
			Oxacillin and tetracycline	*S. aureus* ATCC 33591	X			
			Gentamicin and tetracycline	Methicillin-susceptible *S. aureus* ATCC 6538 (MSSA),	X			
			Gentamycin	*P. aeruginosa* ATCC 27853	X			
			Erythromycin and tetracycline	E. coli ATCC 25922	X			

*Coriander (*Coriandrum sativum*) /Apiaceae , Cinnamomum (Cinnamomum cassia) /Lauraceae, Ziziphora (Ziziphora hispanica*) / *Lamiaceae*		*Cinnamomum cassia* EOs MIC: < 5 mg/ml.			*E. coli, P. aeruginosa, K. pneumoniae* and *P. mirabilis* susceptible and resistant phenotypes	Clinical samples from patients with UTIs	X				Cassia EOs was the most antimicrobial active against all strains	[131]
Eucalyptus (*Eucalyptus camaldulensis*) leaf /Myrtaceae	Patulenol, cryptone, p-cimene, 1,8-cineole, terpinen-4-ol and β-pinene	EOs MIC: 0.5 to 2 µl/ mL	Ciprofloxacin, gentamicin and polymyxin B		MDR *Acinetobacter baumannii*	Wound isolates	X					[20]
Eucalyptus (Eucalyptus globulus) / Myrtaceae					MRSA		X	X		X	Exerted more potent anti-QS activity, even at low concentrations, compared with 1,8-cineole.	[131]
Eucalyptus (*Eucalyptus globulus*), Tea Tree (*Melaleuca alternifolia*) , Clove (*Syzygium aromaticum*) / Myrtaceae, Cinnamomum (*Cinnamomum zeylanicum*) / Lauraceae	1,8 cineole is the predominant component	EOs MIC: 10 mg/ml; 1,8-cineole MIC: 1.25 mg/ml			*P. aeruginosa* and S. aureus	Clinical isolates	X				EOs have shown nearly equal bacteriostatic and bactericidal activity effects agasint GPB and GNB	[35]

Geranium (*Pelargonium graveolens* Ait*)* / Geraniaceae	Citronellol (26.7%) and geraniol (13.4%) and other major compounds included nerol, citronellyl formate, isomenthone, linalool, 10-epi-γ-eudesmol.	EOs MIC *(E. coli*) :3.0-3.75 µl/ml ; EOs MIC (*C. freundii*) : 5.25-5.75 µl/ml ; EOs MIC (Enterobacter strains) :6.25-8.0 µl/ml ; EOs MIC (*P. aeruginosa and P. mirabilis)* :9.25-10.5 µl/ml			*E. coli, C. freundii, E. sakazakii, E. cloacae, P. mirabilis and P. aeruginosa* resistant strains		X		Geranium EO could serve as an effective antimicrobial agent for topical application on wounds, especially those infected with drug-resistant strains; *E. coli* strains were the most susceptible, while *Pseudomonas* and *Proteus* strains were least susceptible.	[133]
Geranium *(Pelargonium graveolens) / Geraniaceae, Rosemary (Rosemary officinalis) / Lamiaceae, and Peppermint (Mentha piperita) / Lamiaceae*		Mint EOs MIC: 2.5-5 µl/ml); Geranium EOs MIC: 5-20 µl/ml;Rosemary EOs MIC: 5-20 µl/ml	Colistin		XDR colistin-resistant and colistin susceptible A. baumannii	Clinical isolates	X	X	Rosemary and pelargonium EOs showed strong synergistic activity with colistin, reducing colistin MIC values by 2- to 32-fold in XDR-*A. baumannii clinical* isolates. At sub-MIC concentrations, they inhibited 48–90% of biofilm formation in *A. baumannii.*	[40]

Ginger (*Zingiber officinale*) / Zingiberaceae	Eudesmol (8.19%), γ-terpinene (7.88 %), a-curcumene (7.28%), alloaromadendrene (6.56%), zingiberene (6.06 %), α-pinene (5.76 %), δ-cadinene (3.84%), elemol (3.39%), farnesal (3.45%), E-β-farnesene (3.57%), neril acetate (2.8%) and β-myrcene (2.94%	EOs MIC (*S. aureus*) : 0.25 mg/ml ; EOs MIC (*S. epidemidis)*: 0.5 mg/ml ; EOs MIC (*E. faecalis*) :1.0 mg/ml		*S. aureus , E. coli , S. epidermidis , Enterococcus faecalis, Enterobacter aerogenes , E. gergoviae, Klebsiella oxytoca , K. pneumoniae , Micrococcus luteus, P. aeruginosa , Salmonella enterica , S. typhi , Serratia marcescens , Vibrio cholerae*		X	The EOs exhibited potent bactericidal effects, eliminating 99.9% of CFU in *S. aureus* and *S. marcescens* within the first hour of exposure at 0.5 and 0.75 mg/mL, respectively. *S. aureus, S. epidermidis,* and *E. faecalis* were the most susceptible strains.	[134]
	9,12-Octadecadienoic acid methyl ester 466 (50.49%) and Hexadecanoic acid methyl ester (38.05%)	EOs MIC: 1 mg/ml; EOs MBC: 2 mg/ml		*P. aeruginosa* producing extended spectrum β-lactamase (ESβL) enzyme	Wound samples of burn patients	X		[135]
			Cefepim	MDR and XDR *E.coli* blaTEM genotypes	Clinical samples from aptients with Urinary tract infection	X		[99]

Lavender (*Lavandula angustifolia*) / Lamiaceae	linalyl anthranilate (38.42%), linalool (34.56%), β-caryophyllene (4.81%), isoborneol (2.57%), cis-beta-farnesene (2.11%), trans-β-ocimene (1.24%), 3-octanone (1.22%), hexyl butyrate (1.08%) and caryophyllene oxide (1.04%).		Piperacillin	MDR *E. coli* J53 R1	X	X		[136]
	Linalyl anthranilate (45.9%) and linalool (34.5%) , β-caryophyllene (2.4%) and borneol (1.9%)	EOs MIC: 10% (v/v); EOs and meropenem MIC 0.63% (v/v)	Meropenem	Carbapenem resistant KPC-producing *K. pneumoniae* BAA-1705 (KPC-KP) and *E. coli* ATCC 25922	X		Out of 31 EOs compounds analyzed, 10 exhibited antioxidant properties, while 4 acted as pro-oxidants. The EOs induced oxidative stress in *K. pneumoniae*, leading to the oxidation of the outer membrane. This oxidative damage facilitated the influx of ROS, LVO, and meropenem into the bacterial cells, resulting in cellular damage and eventual cell death.	[137]

Lavender (*Lavandula angustifolia*) / Lamiaceae, Frankincense (*Boswellia sacra*) / Burseraceae, Myrtle (*Myrtus communis*) / Myrtaceae, Thyme (*Thymus vulgaris*) / Lamiacea, Lemon (*Citrus limon) / Rutaceae*, Oregano (*Origanum vulgare*) / Lamiacea	Lavender EOs: linalyl-butyrate (26.5%), linalool (25%) and other compounds with concentrations ranging between 3%–7%: geranyl butyrate, β-caryophyllene, terpinyl acetate, farnesol, ocimene, 4-carvomenthenol.	EOs aqueous extract MIC (*P. aeruginosa*):6.3%; EOs micellar suspension MIC (*P. aeruginosa*: > 50%. EOs aqueous extract MIC/MBC (*K. pneumoniae*): 6.3% EOs micellar suspension MIC/MBC (*K. pneumoniae*):2.5%		MSSA ATCC 29213, MRSA ATCC 43300, *Enterococcus faecalis* ATCC 29212, *E. coli* ATCC 25922, *K. pneumoniae* ATCC 13883, *P. aeruginosa* ATCC 27853		X		Among the tested extracts, only oregano and lavender aqueous extracts demonstrated bactericidal activity, specifically against *K. pneumoniae*. This selective activity highlights the potential of these extracts as targeted antimicrobial agents.	[138]
Lemongrass (*Cymbopogon citratus*) / Poaceae	Geranial (29.4%), neral (30.4%), caryophyllene (25.4%) and indan-1,3-diol monoacetate (7%).	EOs MIC: 0.0781%		*Staphylococcus aureus*		X	X		[96]
	Myrcene is the predominant component			*Klebsiella pneumoniae*, *Pseudomonas aeruginosa* and *Staphylococcus epidermidis*	Patients with Chronic rhinosinusitis	X	X		[139]

Lemongrass (*Cymbopogon citratus*) / Poacea, Clove (*Eugenia caryophyllata*) /Myrtaceae, Salvia (*Salvia sclarea*) /Lamiaceae, Cinnamon (*Cinnamomum zeylanicum*)/ Lauraceae, Citronella (*Cymbopogon nardus*) /Poacea , Eucalyptus (*Eucalyptus globulus*) /Myrtaceae, Fennel (*Foeniculum vulgare*), Spearmint (*Mentha spicata*) /Lamiaceae, Peppermint (*Mentha piperita*) /Lamiaceae , Lemon (*Citrus limon*) Rutaceae, and Thyme (*Thymus vulgaris*) /Lamiaceae, Rosemary (*Rosmarinus officinalis*) /Lamiaceae	Citral (60.6%) , geranial (34.5%) and neral (26.1%). Its minor volatile compounds were caryophyllene-oxide (4.7%), geraniol (4.3%), linalool (2.6%), α-terpineol (1.7%), γ-cadinene (1.4%) and limonene (1.3%)	EOs MIC/ MBC: 0.1 mg/ml		*Bacillus thuringiensis, Kytococcus sedentarius, Dermatophilus congolensis*	Pitted keratolysis (i.e., bacterial skin infection mostly affecting the pressure-bearing areas of the soles) lesions	X		[44]

Lemongrass (*Cymbopogon citratus*) /Poaceae, Lavender (*Lavandula angustifolia*) /Lamiaceae, Marjoram (*Origanum majorana*) /Lamiaceae, Peppermint (*Mentha x piperita*) /Lamiaceae, Tea Tree (*Melaleuca alternifolia*) /Myrtaceae, and Rosewood (*Rosmarinus officinalis*) /Lamiaceae	The majority of EOs constituents were monoterpenes, including: monoterpene alcohols such as terpinen-4-ol or geraniol; bicyclic monoterpenes such as camphene, borneol, pinenes, sabinene or camphor; acyclic monoterpenoids (or derivatives) such as myrcene, geranyl acetate, citronellol or linalool. Several sesquiterpenes such as cadinene, caryophyllene, caryophyllene oxide or globulol were also present. Some compounds such as pinene, limonene, citrol, dihydrolinalool, α-terpineol, terpiene-4-ol, p-cymene, myrcene and 1,8-cineole were present in more than one oil.	Terpinen-4-ol MIC: 0.125-0.5% v/v; Geraniol MIC: 0.125-1% v/v			MDR *Burkholderia cepacia* complex	Patients with cystic fibrosis	X		[140]
Peppermint (*Mentha x piperita*) /Lamiaceae	Menthol (43.66%), menthone (20.02%), iso-menthone (7.73%), 1.8-cineole (6.49%) and menthyl acetate (3.31%)				*S. aureus* , *Listeria monocytogenes, Salmonella enterica* , *E. coli* and *P. aeruginosa*		X		[154]

	Menthol (68%), Isomenthone (9.48%), Menthone (8.36%), Limonene (1.85%)	EOs MIC: 20–40 mg/Ml; EO and gentamycin MIC (*B. subtilis*): 0.5 to 0.01 µg/ml; EOs MIC *(P.aeruginosa*) : 2 to 0.06 µg/ml; EOs MIC (*K.pneumoniae*) : 32 to 1 µg/Ml; EOs MIC reduced 16-fold for *A. baumannii*	Gentamycin	Bacillus cereus ATCC 10876, Bacillus subtilis ATCC 6633, Staphylococcus aureus ATCC 6538p, Staphylococcus aureus ATCC 29213, Staphylococcus aureus ATCC 43300 (MRSA), Enterococcus faecalis ATCC 29212, Escherichia coli ATCC 25922, Klebsiella pneumoniae ATCC 19883, Acinetobacter baumanni ATCC 19606, Pseudomonas aeruginosa ATCC 27853.	X		*M. piperita* EOs showed strong synergistic activity with gentamicin, reducing the MIC by over 30-fold for six out of ten tested bacterial strains.	[154]
	Menthol (50.1435%), followed by menthone (24.4276%), menthyl acetate (6.1701%), eucalyptol (5.7894%), cis-β-Terpineol (1.8929%), o-cymene (1.0384%) and caryophyllene oxide (1.0148%	EOs MIC: 8%; EOs and meropenem MIC : 1%	Meropenem	MDR *E. coli* pMG309 harboring a plasmid encoding β-lactamase, KPC-3	X	X		[141]

	Monoterpene hydrocarbons(34.23%), monoterpene oxygenates (60.83%), sesquiterpene hydrocarbonates (4.63%) and sesquiterpene oxygenate (0.21%)	EOs MIC (*S. aureus, E. coli, and P. mirabilis*) :20 mg/ml; EOs MIC *(K. pneumoniae, P. aeruginosa, and A. baumannii*): 40 mg/ml ; MBC= MIC for most strains, except for *P. aeruginosa*; EOs MBC was twice the MIC.		MDR *A. baumannii, E. coli, K. pneumoniae, P. mirabilis, P. aeruginosa* and *S. aureus.*	A. baumannii : bronchoalveolar lavage samples; E. coli: urine and wound secretion; K. pneumoniae: urine and bronchoalveolar lavage; P. mirabilis: wound secretion; P. aeruginosa: wound secretion and otic discharge; S. aureus: nasal swabs and wound secretion.	X		Exhibited a bactericidal activity of peppermint EOs on all tested MDR or XDR GPB and GNB.	[142]
Peppermint *(Mentha piperita*) /Lamiaceae, Cinnamon (*Cinnamomum Verum*) / Lauraceae, Tea Tree (*Melaleuca alternifolia*) /Myrtaceae, Lavender (*Lavandula Angustifolia*) /Lamiaceae, Lemongrass *(Cymbopogon citratus*) /Poaceae				*S. aureus* and *K. pneumoniae*		X	X	At a concentration of 30 µl/100 µl, cinnamon EOs exerted the highest antibiofilm activity followed by peppermint EOs at the same concentrations	[143]

Mint (*Mentha spicata*) /Myrtaceae	Carvone (78.76%), limonene (11.50%), β-bourbonene (11.23%), cis-dihydrocarveol (1.43%), trans-caryophyllene (1.04%), menthone (1.01%), menthol (1%), and terpinen-4-ol (0.99).	EOs MIC/ MBC (L. monocytogenes) : 2.5 µL/ml		*S. aureus, Bacillus subtilis, Bacillus cereus, Listeria monocytogenes, Salmonella typhimurium, and E. coli* O157:H7		X		GPB were more susceptible to *M. spicata* EOs than GNB.	[12]
	Carvone (49.5%), limonene (16.1%), 1,8-cineole (8.7%), *cis*-dihydrocarvone (3.9%), β-caryophyllene (2.7%), germacrene D (2.1%) and β-pinene (1.1%)	EOs MIC (*S. epidermidis*) :32 µg/ml		*S. epidermidis* ATCC 12228 and *E. coli* ATCC 29425		X			[144]
Menthol and Mint (*Mentha longifolia)*			Imipenem; Ciprofloxacin	Carbapenem-resistant and fluoroquinolones-resistant *A. baumannii*	Clinical isolates	X	X	Menthol combined with imipenem reduced the MIC of imipenem by 16-fold. *Mentha longifolia* EOs reduced the MICs of ciprofloxacin and imipenem by 4-fold and 8-fold, respectively, demonstrating strong synergistic activity with these antibiotics.	[192]
Oregano EOs (*Origanum vulgare*) /Lamiaceae	Carvacrol (71%), γ-terpinene (4.5%), β-caryophyllene (4%), p-cymene (3.5%), and thymol (3%).	EOs MIC (*K. pneumoniae* and *S. marcescens*) : 0.059% (v/v) ; *EOs MIC (A. baumannii):* 0.015 % (v/v).		Carbapenem-resistant *K. pneumoniae and S. marcescens, and A. baumannii*	rectal swab, urine sample, and nasal swab, respectively	X			[298]
	Carvacrol (71.0%), β-caryophyllene (4.0%), γ-terpinene (4.5%), p-cymene (3,5%), and thymol (3.0%).	EOs MIC: 1.75 to 3.50 mg/ ml	Polymyxin B	MDR *A. baumannii*	Clinical isolates	X			[192]

Oregano and thyme red /Lamiaceae	Carvacrol and thymol are the predominant components		Uropathogenic *E. coli* O6:H1 strain CFT073 (ATCC 700928)	Clinical isolates	X	X		Oregano and thyme red EOs demonstrated potent antibiofilm activity, inhibiting UPEC biofilm formation by more than 60% at a concentration of 0.01% (v/v). Notably, these oils did not significantly inhibit planktonic cell growth, with less than 15% inhibition observed at OD620, compared with untreated controls. This selective activity suggests their potential as targeted antibiofilm agents without affecting bacterial viability.	[32]
Oregano (*Origanum compactum*)	Carvacrol, thymol, p-cymene and γ-terpinene. The percentage of carvacrol (24.71%) and thymol (15.32%)at vegetative stage and carvacrol (43.584%), thymol (10.33%) at the flowering stage.	MIC and MBC values ranged between 0.5% and 0.031% (v/v); MIC = MBC = 0.0 312% (v/v) against *B. subtilis*	*E.coli* and *B. subtilis*		X	X	X	The antibacterial activity of *O. compactum* essential oils is attributed to their ability to disrupt cell membrane integrity and increase membrane permeability. This disruption leads to the leakage of genetic materials (DNA and RNA), ultimately causing bacterial cell death.	[196]
Oregano (*Origanum onites*) /Lamiaceae	Carvacrol (51.4%), linalool (11.2%), p-cymene (8.9%) and γ-terpinene (6.7%)	MIC:1.56–25 µl/ml	Extended spectrum beta lactamase (ESBL) producer, carbapenem resistant *E. coli*	Clinical samples	X				[148]
Oregano EOs /Lamiaceae	Carvacrol (72.25%), thymol (6.62%), p-cymene (5.21%), γ-terpinene (4.12%), and α-pinene (1.21%)	MIC: 0.08 to 0.64 mg/ml	*A. baumannii, P. aeruginosa, and MRSA*	Clinical samples	X	X			[197]

Wild oregano /Lamiaceae , Garlic /Amaryllidaceae , Black pepper /Piperaceae		Oregano EOs MIC: 0.02-1.25 mg/ml; Garlic EOs MIC: 0.02-40 mg/ml; Black pepper EOs MIC: 0.04-40 mg/ml			*Clostridioides difficile*	Stool specimens of hospitalized patients with diarrhea and CDI	X		[120]
Oregano (Origanum vulgare) /Lamiaceae, Thyme (Thymus vulgaris) /Lamiaceae , Lavender (Lavandula angustifolia) /Lamiaceae, Peppermint (Mentha x piperita) /Lamiaceae, Tea Tree (Melaleuca alternifolia) / Myrtaceae	Carvacrol is the predominant component of the EOs	Thyme and oregano EOs MIC:256 to 512 μg/ml; Carvacrol EOs MIC: 64 to 256 μg/ml	Erythrmoycin and carvacrol		*Erythromycin-resistant Streptococcus pyogenes [Group A streptococci (GAS)*	Children with pharyngotonsillitis	X		[210]
Rosemary (*Rosmarinus officinalis*) /Lamiaceae	1,8-cineole (17.16 %), α-pinene (16.95 %) and verbenone (15.78 %)	MIC=MBC: 0.06 to 0.16 ± 0.07 mg/ml			*S. aureus (ATCC 29737), K. pneumoniae (ATCC 10031), and Proteus vulgaris (PTCC 1182)*	Urine samples from patients suspected of UTI	X		[198]

Rosemary (*Rosmarinus officinalis*) /Lamiaceae, Oregano (*Origanum majorana) /Lamiaceae*, Thyme (*Thymus zygis*) /Lamiaceae, Juniper (*Juniperus communis*) Cupressacea, Ginger (*Zingiber officinale*) /Zingiberaceae	*J. communis* EOs: α-Pinene (47.1%), β-Myrcene (11.7%) and Limonene (6.2%); Z. officinale: α-Zingiberene (33.1%), β-Sesquiphellandrene (13.5%), ar-curcumene (8%), β-Bisabolene (6.4%), Camphene (7.4%) and Limonene (5.7%); *O. majorana* EOs: terpinen-4-ol (25.9%), γ-Terpinene (16.9%), Linalool (10.9%), Sabinene (8%) and α-Terpinene (7.7%);T. zygis: Linalool (39.7%), Terpinen-4-ol (11.7%), β-Myrcene (8.6%) and γ-Terpinene (7.6%). *R. officinalis EOs*: 1,8-Cineole (47.7%), α-Pinene (11.7%), Camphor (9.6%) and β-Pinene (6.3%).	Rosemary EOs MIC: 1.56 - 3.125 mg/ml; MBC: 12.5 mg/ml; Thyme EOs MIC: 0.19 - 0.78 mg/ml; MBC: 1.56 - 6.25 mg/ml; Marjoram EOs MIC: 0.19 - 0.78 mg/ml; MBC: 1.56 - 12.5 mg/ml	*E. coli*	Urine samples were from patients with clinical symptoms of UTI	X	X		Rosemary EOs exhibited the highest antibiofilm activity, followed by Thyme and MarjoramEOs; In contrast, Thyme EOs demonstrated the strongest antibacterial activity against *E. coli* isolates, outperforming Marjoram and Rosemary EOs; Rosemary EOs showed the lowest antibacterial activity, requiring higher concentrations to achieve both inhibitory and bactericidal effects.	[168]
Rosemary (Rosmarinus officinalis) /Lamiaceae , Tea Tree (Melaleuca alternifolia) /Myrtaceae	The predominant components in Rosemary EOs: Eucalyptol and α-pinene; Tea Tree EOs: 4-terpineol and terpinolene.	Rosemary EOs MIC: 0.5 mg/ml; Tea Tree EOs MIC: 0.25 mg/ml	*S. aureus* (ATCC 25923) and *E. coli* (ATCC 25922)		X	X		Tea Tree EO was more inhibitory than Rosemary EO for biofilm formation by the two test strains.	[199]
Tea Tree (*Melaleuca alternifolia*) /Myrtaceae	terpinen-4-ol (40.4%), γ-terpinene (19.5%), and α-terpinene (7.7%), 1,8-cineole (5.2%), p-cymene (4.7%), α-terpineol (3.3%), and α-terpinolene (3.1%).	EOs MIC (S. aureus) : 0.048–3.125 mg /mlfor the whole essential oil,; terpinen-4-ol MIC (S. aureus): 0.048–1.52 mg/ml; EOs MBC: 25 to 50 mg/ml; terpinen-4-ol	MRSA	Superficial and deep pus , blood culture and strains of various other specimens (tracheal aspiration, wound).	X	X	X		[187]

		MBC (S. aureus): 6.25–50 mg/ml							
			Tobramycin	MDR *P. aeruginosa*	Patients with cystic fibrosis	X	X	The TTO antibiofilm properties and synergistic relationship with tobramycin against CFPA *in vitro* make the inhaled EOs a promising candidate as a potential therapeutic agent.	[130]
	α-pinene, α-terpineol , and p-cymene, α- and γ-terpinene			MRSA (ATCC 33592), MDR P. aeruginosa (ATCC BAA-2108)		X	X		[181]
				Carbapenem-resistant *S. marcescens*		X		Tea Tree EOs nanoemulsion was reduced the colonization of *CRE-S. marcescens* in a *C. elegan*s in *in vivo* model.	[155]
	Terpinen-4-ol (42.3%), γ-Terpinene (19.4%), α-Terpinene (8.5%)			*S. aureus* ATCC 6538		X	X	TTO incorporated into the base hydrogel 1% demonstrated antimicrobial efficacy *in vitro* and in biofilm control in an *ex vivo* model; TTO inhibited *S. aureus in vitro, in vivo* and *ex vivo*.The EOs Hydrogel reduced the severity of *S. aureus*-infected wounds	[299]

	terpinen 4-ol (35.4%), eucalyptol(15.2%,, α-pinene(12.4%), and γ-terpinene (9.8%).		CFZ/OXA/AMK against both MSSA and MRSA and with MEM/AMK/COL against all Gram-negative microorganism	MSSA, E. coli, and clinical strains of methicillin-resistant S. aureus (MRSA), extended-spectrum beta lactamases producer carbapenem-sensitive Klebsiella pneumoniae (ESBL-CS-Kp), carbapenem-resistant K. pneumoniae (CR-Kp), Acinetobacter baumannii (CR-Ab), and Pseudomonas aeruginosa (CR-Pa).		X		TTO showed a potent bactericidal activity against all the tested microorganisms; TTO in combination with each reference antimicrobial showed a high level of synergism at sub-inhibitory concentrations, particularly with oxacillin (OXA) against MRSA.	[161]
Oregano (*Thymbra capitata*) /Lamiaceae, Thyme (*Thymus pallescens*) /Lamiaceae, White Wormwood (*Artemesia herba-alba*) /Asteraceae		T. pallescens EOs MIC: 0.16 to 0.63 mg/ml		*K. pneumoniae, E. coli,* and *S. aureus.*	Clinical isolates	X	X	*T. pallescens* EOs was the most effective antimicrobial agent against pathogenic bacteria, with large zones of inhibition (up to 50 mm against S. aureus), low MICs (0.16 to 0.63 mg/mL), and powerful biofilm eradication up to 0.16 mg/mL in both 24 h and 60-min exposure times.	[200]

Thyme (*Thymus daenensis* L.) , Oregano (*Origanum vulgare* L.) /Lamiaceae	Thyme EOs: Carvacrol (40.69%), γ - terpinene (30.28%) and α -terpinene (5.52%) .Oregano EOs: pulegone (44.31%), 1,8 - cineole (17.47%), and borneol (6.20%)	Thyme EOs MIC: 0·625–2·5 µl/ml; Oregano EOs MIC: 1·25–5 µl/ ml		Fluoroquinolone-resistant *Streptococcus pneumoniae*	Clinical isolates	X	X	X		*Thymus daenensis* and *Origanum vulgare* EOs have strong antimicrobial and anti-efflux pump activity against *S. pneumoniae* clinical isolates and might be useful in controlling pneumococcal infections.	[136]
Thyme (*Thymus daenensis*) , Summer savory (*Satureja hortensis*) , Oregano (*Origanum vulgare*) /Lamiaceae	Thymol, carvacrol, p-cymene, pulegone, and 1,8-cineole	Thyme EOs MIC: 0.625–1.25 µl/ml; Satureja hortensis EOs MIC:2.5 µl/ml; Oregano EOs MIC: 2.5–10 µl/ml; Thyme EO MBC: 1.25–2.5 µl/ml; Satureja hortensis EOs MBC:2.5–5 µl/ml; Oregano EOs MBC: 5–20 µl/mll		*S. pneumoniae NCTC 7466*	Clinical isolates	X	X		X		[137]
*Thyme (Thymus vulgaris) /Lamiaceae, Cinnamon (*Cinnamomum verum*) /Lauraceae, Oregano (*Origanum majorana*) /Lamiaceae, and Clove (Eugenia caryophyllata) /Myrtaceae*				MDR bacteria	Clinical isolates	X	X				[201]

Thyme (Thymus vulgaris) /Lamiaceae, Clove (Eugenia caryophyllata) /Myrtaceae, Oregano (Origanum vulgare) /Lamiaceae, Rosemary (Rosmarinus officinalis) /Lamiaceae, Lavender (Lavandula officinalis) /Lamiaceae, Tea Tree (Melaleuca alternifolia) /Myrtaceae	Oregano EOs: carvacrol (71.8%) and the monoterpene hydrocarbon p-cymene. Rosemary EOs: 0,8-cineole (43.9%), α-pinene; Thyme EOs: p-cymene (47.9%) and the oxygenated monoterpene thymol (43.1%); CloveEOs: eugenol (85%) and the sesquiterpene hydrocarbon β-caryophyllene (9%); Tea Tree EOs: 4-terpineol (39.9%), γ-terpinene (14.4%) and α-terpinene (8.8%).	*B. cepacia* complex	Patients with cystic fibrosis	X			[153]
Black pepper (*Piper nigrum) /Piperaceae* , Cananga (*Cananga odorata) /Annonaceae*, Myrrh (*Commiphora myrrha)* /Burseraceae	Cis-nerolidol is thecommon constituent	*S. aureus* strain ATCC 6538		X	X	Black pepper, cananga, and myrrh oils and their common constituent cis-nerolidol at 0.01 % markedly inhibited *S. aureus* biofilm formation; Black pepper oil down-regulated the expressions of the α-toxin gene (hla), the nuclease genes, and the regulatory genes. In addition, black pepper, cananga, and myrrh oils and cis-nerolidol attenuated *S. aureus* virulence in the nematode Caenorhabditis elegans.	[202]

Black pepper (*P. nigrum* (black and white pepper), *P. cubeba,* and *P. longum*) / Burseraceae	Piper nigrum EOs: monoterpenes (87.6%), sesquiterpenes (12.3%); White pepper EOs: monoterpenes (80%) , sesquiterpenes (19.6i%), in addition to α-pinene, β-pinene, 3-carene, limonene, and β-caryophyllene; *Piper longum* EOs sesquiterpenes (50%), alkanes (18.2%) and alkene (13.6%); *Piper cubeba* EOs aromatics (59%), with eugenol (10.7%) and methyl eugenol (47.4%) representing the major components along with β-myrcene (21.2%) and 1,8-cineole (6.4%).	*P. longum* EOs MIC: 1.95 µg/ml; White pepper EOs MIC =:3.90 µg/ml; *P. cubeba* and *P. nigrum* EOs MIC : 7.81 µg/ml.		*Helicobacter pylori*	X		*P. longum* EOs was the most efficient anti-Helicobacter activity with an MIC value similar to clarithromycin MIC	[178]
Laurel (*Nectandra megapotamica)* / Lauraceae	Caryophyllene oxide (22.3%), spathulenol (12.7%), α-copaene (8.1%), αcadinol (7.3%) and globulol (6.0%).	EOs MIC: 36000 µg/ml	Imipenem	MDR OXA-23-producing *A. baumannii*	X	X		[204]
Frankincense Tree (Boswellia sacra)	α-pinene > 50% ,Camphene, (+)-3-carene and 1-limonene	EOs MIC (*E.coli*) : 15,600 µg/Ml		*S. aureus* (NCTC 6571), *Bacillus spp., E. coli* (NCTC 10418), *P. aeruginosa* (NCTC 10662)	X		Frankincense EOs smoke showed antimicrobial activity against *S. aureus, E. coli* and airborne bacteria	[116]

Commiphora species (*C. myrrha, C. confusa, C. baluensis, C. pseudopaolii, C. guidotti, C. holtziana and C. kataf*) /Burseraceae	α-Pinene (5.6–8.1%), β-caryophyllene (nd-16.5%), cis-β-ocimene (nd-7.4%), α-santalene (nd-40.8%), α-bourbonene (6.9–37.4%), β-elemene (nd-26.2%), curzerene (4.5–8.2%), p-cymene (1.8–54.5%), curzerenone (1.6–25.5%), (Z)-α-bisabolene (nd-10.4%), β-selinene (nd-9.8%), germacrene D (nd-8.4%), cresol methyl ether (nd-8.5%), 7-epi-sesquithujene (nd-8.8%), dehydro-aromadendrene (nd-36.9%), δ-elemene (nd-31.1%) and spathulenol (0.2–10.4%)	EOs MIC : 0.06–8.0 mg/ml.		*S. aureus* ATCC126000, *Enterococcus faecalis* ATCC29212, *E. coli* ATCC8739, *Moraxella catarrhalis* ATCC23246	X	X	*C. holtziana* and *C. myrrha* exhibited the best anti-qQS sensing activity by inhibiting violacein production.	[205]

Tea Tree (Melaleuca alternifolia) /Myrtaceae, Sage (Salvia officinalis) /Lamiaceae, Thyme (Thymus vulgaris) /Lamiaceae, and Eucalyptus sp. /Myrtaceae	Alpha- and beta-thujone; 1,8-cineole; camphor; and humulene are among most identified compounds in *Salvia officinalis* EOs. Thyme EOs include thymol, carvacrol, and paracymene. The antibacterial activity of Eucalyptus extracts is due to components such as 1,8-cineole, citronellal, citronellol, citronellyl acetate, p-cymene, eucamalol, limonene, linalool, β-pinene, γ-terpinene, α-terpinol, alloocimene, and aromadendrene. Tea Tree EOs consists of about 100 terpinenes and their alcohols, of which terpinene-4-ol, α-pinene, linalool, and α-terpineol	Tea Tree EOs MIC: 0.538 to 0.759%;Thyme EOs MIC: 0.246–0.470%; Sage EOs MIC: 4.583–5.000%; Eucalyptus EOs MIC: 2.361–3.214%.		*P. aeruginosa* resistant strains	Clinical isolates	X	X		X	Tea Tree and thyme EOs are particularly effective against *P. aeruginosa* strains, regardless of their isolation source and antibiotic resistance. These EOs showed much-reduced MIC values and were able to inhibit biofilm formation when utilized in sub-inhibitory concentrations.	[128]

Cinnamon bark (*Cinnamomun zeylanicum*) /Lauraceae, Clove (*Syzygium aromaticum*) /Myrtaceae, Lemongrass (*Cymbopogon flexuosus*) /Myrtaceae, Oregano (*Origanum vulgare*) /Lamiacea, Rosemary (*Rosmarinus officinalis*) / Lamiacea, Thyme (*Thymus vulgaris*) /Lamiacea, Tea Tree (*Melaleuca alternifolia*) /Myrtaceae, Manuka (*Leptospermum scoparium*) /Myrtaceae, and Thieves (a proprietary blend of cinnamon, clove, lemon, eucalyptus, and rosemary)	Cinnamon bark EOs:trans-cinnamaldehyde 68.4%, eugenol 8.5%, cinnamyl acetate 4.1%; Clove EOs: eugenol 78.9%, eugenyl acetate 13.0%, trans-beta-caryophyllene 6.5%; Lemongrass EOs :geranial 39.2%, neral 32.0%, geraniol 6.9%; Manuka EOs:Leptospermone 16.4%, calamenene 12.8%, isoleptospermone 6.1%; Oregano EOs:carvacrol methyl ether 67.4%, para-cymene 7.7%, gamma-terpinene 4.8%; Tea Tree EOs: terpinene-4-ol 37.1%, gamma-terpinene 20.8%, alpha-terpinene 11.3%; Thieves EOs: eugenol 35.5%, limonene 16.9%, trans-cinnamaldehyde 12.7%; Thyme EOs: thymol 43.3%, para-cymene 16.0%, gamma-terpinene 7.7%.		Extended-spectrum beta-lactamase-producing Enterobacteriaceae, carbapenem-resistant Enterobacteriaceae, MDR P. aeruginosa, and MRSA	Clincal isolates	X		Oregano, thyme, cinnamon bark, and lemongrass EOs exerted the largest zones of inhibition against GPB , whereas cinnamon bark had the largest zone of inhibition against *P. aeruginosa*. Oregano, thyme, and cinnamon bark EOs had the largest zones of inhibition against Enterobacteriaceae.	[209]
Sage *(Salvia fruticosa, Salvia officinalis and Salvia sclarea) /* Lamiaceae		tetracycline	Tetracycline resistant *S. epidermidis*	Clinical isolates	X	X		[195]

Basil (*Ocimum basilicum)*, Sage (*Salvia officinalis*) / Lamiaceae	Basil EOs: Linalool and (E)-anethole; Sage EOs: α-thujone and camphor				*P. aeruginosa* resistant strains	Urine sample, skin, throat, eye, ear, and wound swabs	X	X		[189]
Basil (*Ocimum basilicum)*,Lamiaceae	Basil EOs MIC : 1024 µg/ml	Imipenem (MIC = 4 mg/mL) and ciprofloxacin (MIC = 2 mg/mL)		*S. aureus and P. aeruginosa*	Clinical samples	X				[189]
Pituranthos chloranthus, *Teucruim ramosissimum, Mastic (Pistacia lentiscus*) areal parts /Apiaceae	Sabinene, limonene, terpinen-4-ol, and β-eudesmol.	*Pistacia chloranthus* EOs MIC:0.25-0.5 mg/ml; *Teucrium ramosissimum* EOs MIC: 0.25-1 mg/ml; *Pistacia lentiscus* EOs MIC: 0.125-1 mg/ml against MRSA. MIC: 1 mg/mL against *E. coli and Acinetobacter baumannii*	Ofloxacin and novobiocin against ESBL-producing *E. coli*		E. coli (ESBL), ceftazidime-resistant A. baumannii, and MRSA	Patients with UTI	X		MRSA demonstrated notable susceptibility to the tested EOs; *E. coli* and *A. baumannii* were more resistance to all tested EOs	[296]

Caraway (*Carum c*arvi L.) /Apiaceae , Fennel (*Foeniculum vulgare* Mill.) /Apiaceae , Peppermint (*Mentha × piperita* L.) /Apiaceae , Geranium (*Pelargonium graveolens* L'Hér.) /Geraniaceae, Basil (*Ocimum basilicum* L.) /Apiaceae , Clove (*Syzygium aromaticum*) / Myrtaceae, Thyme (*Thymus vulgaris*) /Apiaceae , Sage (*Salvia sclarea*) /Apiaceae , Lavender (*Lavandula angustifoli*a) /Apiaceae			Peppermint EOs and gentamicin; Caraway EOs and gentamicin	Gentamicin combined with thyme, fennel, basil, and clary sage EOs.	ESBL and New Delhi metallo-β-lactamase-1 (NDM-1) producing *K. pneumoniae*		X	Peppermint EOs and gentamicin induced synergistic effects against all tested strains; Caraway EOs and gentamicin induced synergistic effects against ESBL-strains and gentamicin-resistant strains.	[89]

Mexican oregano (*Lippia origanoides) /Verbenaceae*, thymol–carvacrol II chemotype), Thyme (*Thymus vulgaris*) /Verbenaceae	*Lippia origanoides*, thymol–carvacrol II chemotype EOs:γ-terpinene (5.2%), p-cymene (1.1%), thymol (32.7%), carvacrol (18.8%), and trans-β-caryophyllene (6.4%);Thyme EOs: γ-terpinene (9.5%), p-cymene (20%), linalool (4.7%), trans-β-caryophyllene (9.5%), and thymol (23%).	*Lippia origanoides* thymol–carvacrol II chemotype EO MIC 0.45 mg/ml; Thyme EOs MIC :0.75 mg/ml		*E. coli* ATCC 25922 and *S. aureus* ATCC 29213		X	X		X		[186]
Lemon verbena (*Lippia alba*) /Verbenaceae	Limonene, neral, carvone, geraniol, bicyclosesquitelandrene, geranial, piperitenone, β-bourbonene, and trans-β-caryophyllene.			S. aureus ATCC 25923		X			X	*L. alba* EOs containing geranial/neral showed anti-QS activity against *C. violaceum* and antibacterial activity against S. aureus.	[188]
Java plum *(Syzygium cumini)* leaves /Myrtaceae	α-pinene (53.21%) was the major consituent	EOs MIC (*E. coli*): 512 µg/ml		*E. coli* ATCC 25922, *P. aeruginosa* ATCC 25853 and *S. aureus* ATCC 25923 and MDR *E. coli* , *P. aeruginosa* and *S. aureus*	clinical isolates	X				The oil showed moderate activity against *E. coli* ATCC 25922 only and potentiated the effect of antibiotics demonstrating possible synergism when associated with gentamicin, erythromycin and norfloxacin against *E. coli* and *S. aureus* .	[210]
Immortelle EOs /Asteraceae	Neryl acetate (21.2%), however, α-curcumene (15.9%)	EOs MIC (*Haemophilus spp.*): 0.312 mg/ml		*Haemophilus influenzae* DSM 4690, *H. parainfluenzae* DSM 8978, *P. aeruginosa* ATCC 27853, *S. pneumoniae* DSM 20,566		X	X			Immortelle EOs has antibacterial and anti-biofilm effects against respiratory tract bacteria used in this study. *H. parainfluenzae* was the most sensitive to each treatment, while *P.aeruginosa* was the most resistant.	[211]

Camellia *(Camellia japonica) seeds /Theaceae*	α-terpineol, α-terpinolene, α-pinene, β-pinene, α-terpenyl acetate, spathulenol	MIC=MBC: 50 µg/ml, with concentration-dependent inhibition observed (10% at 5 µg/mL, increasing to 52% at 30 µg/mL)			Carbapenemase production *K. pneumoniae*	Patients with UTI	X				[212]
Pennyroyal (*Mentha pulegium*) /Lamiaceae, White Wormwood (*Artemisia herba alba*) / Asteraceae	*M. pulegium* EOs:pulegone (74.8%) and neoisomenthol (10.0%); A. herba *alba* EOs: camphor (32.0%), α-thujone (13.7%), 1,8-cineole (9.8%), β-thujone (5.0%), bornéol (3.8%), camphene (3.6%), and p-cymene (2.1%).	*M.pulegium* EOs MIC: 1.2 to 9.4 µl/ml; *A. herba alb* EOs MIC : 1.2 to 4.7 µl/ml.	*M. pulegium* EOs and amikacin against imipenem-resistant *Acinetobacter baumannii* ; *A. herba alba* EOs with cephalexin against MRSA.		*Listeria innocua, S. aureus ATCC25922, and MRSA; E. coli ATCC25922, P.aeruginosa ATCC27853, and Imipenem-resistant A. baumannii* , producing OXA-23 enzyme and resistant to cefotaxime (CTX) and cefepime (FEP).	Listeria innocua, S. aureus ATCC25922, and MRSA were isolated from pus; E. coli ATCC25922, P.aeruginosa ATCC27853, and Imipenem-resistant A. baumannii isolated from the catheter.	X			M. pulegium EOs and amikacin; A. herba alba EOs and cephalexin.	[177]
Tea Tree *(Melaleuca alternifolia) /Theaceae,* Eucalyptu*s (Eucalyptus globulus) / Theaceae,* Peppermint *(Mentha x piperita) /Lamiacea,* and Thyme *(Thymus vulgaris) /Lamiacea*		Tea Tree EOs MIC: 0.5 - 4 µg/ml for *K. pneumoniae* (55%), *P. aeruginosa* (45%), and *E. coli* (95%) Thyme EOs MIC: 1 - 16 µg/ml for *K. pneumoniae* (90%), *P. aeruginosa* (90%), and *E. coli* (85%) Peppermint EOs MIC: 8 - 128 µg/ml for *K.*			*ESBL E. coli* and *K. pneumoniae*, metallo-beta-lactamase (MBL)-producing *P. aeruginos*a and carbapenemase (KPC)-producing *K. pneumoniae.*	Urine, rectal swabs, and respiratory tracts	X	X			[32]

pneumoniae (90%), *P. aeruginosa* (80%), and *E. coli* (95%)<br>Eucalyptus EOs MIC: 32 - 64 µg/ml for *K. pneumoniae* (90%), *P. aeruginosa* (80%), and *E. coli* (95%)
